# Cation Substitution Strategy for Developing Perovskite Oxide with Rich Oxygen Vacancy-Mediated Charge Redistribution Enables Highly Efficient Nitrate Electroreduction to Ammonia

**DOI:** 10.1021/jacs.3c06402

**Published:** 2023-09-20

**Authors:** Kaibin Chu, Wei Zong, Guohao Xue, Hele Guo, Jingjing Qin, Haiyan Zhu, Nan Zhang, Zhihong Tian, Hongliang Dong, Yue-E. Miao, Maarten B. J. Roeffaers, Johan Hofkens, Feili Lai, Tianxi Liu

**Affiliations:** † The Key Laboratory of Synthetic and Biological Colloids, Ministry of Education, School of Chemical and Material Engineering, International Joint Research Laboratory for Nano Energy Composites, Jiangnan University, Wuxi 214122, China; ‡ Department of Chemistry, KU Leuven, Celestijnenlaan 200F, Leuven 3001, Belgium; § Engineering Research Center for Nanomaterials, Henan University, Kaifeng 475004, China; ∥ Center for High Pressure Science and Technology Advanced Research, Shanghai 201203, China; ⊥ State Key Laboratory for Modification of Chemical Fibers and Polymer Materials, College of Materials Science and Engineering, Donghua University, Shanghai 201620, China; # cMACS, Department of Microbial and Molecular Systems, KU Leuven, Celestijnenlaan 200F, Leuven 3001, Belgium; ¶ Max Planck Institute for Polymer Research, Ackermannweg 10, Mainz 55128, Germany; ∇ John A. Paulson School of Engineering and Applied Sciences, Harvard University, Cambridge, Massachusetts 02138, United States

## Abstract

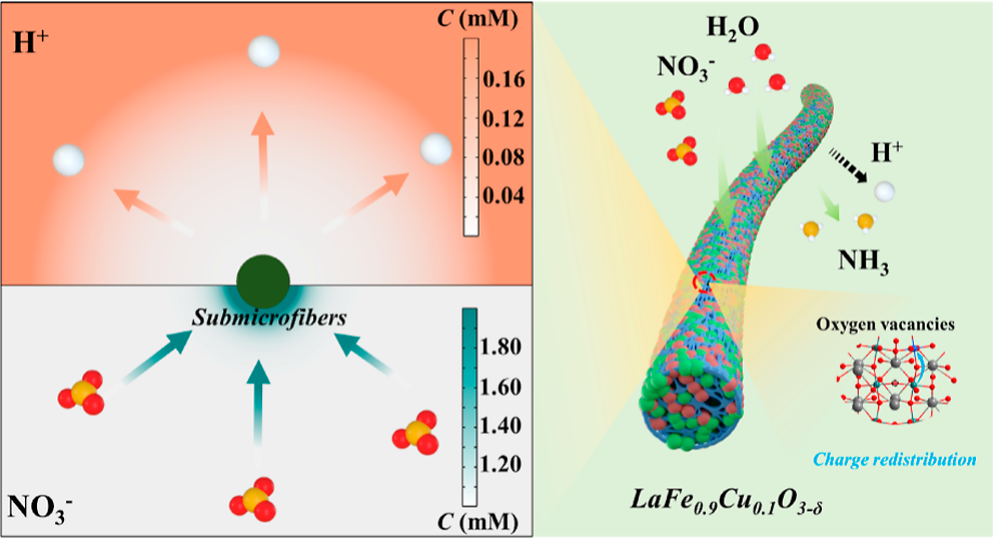

The electrocatalytic nitrate (NO_3_^–^) reduction reaction (eNITRR) is a promising method for ammonia synthesis. However, its efficacy is currently limited due to poor selectivity, largely caused by the inherent complexity of the multiple-electron processes involved. To address these issues, oxygen-vacancy-rich LaFe_0.9_M_0.1_O_3−δ_ (M = Co, Ni, and Cu) perovskite submicrofibers have been designed from the starting material LaFeO_3−δ_ (LF) by a B-site substitution strategy and used as the eNITRR electrocatalyst. Consequently, the LaFe_0.9_Cu_0.1_O_3−δ_ (LF_0.9_Cu_0.1_) submicrofibers with a stronger Fe–O hybridization, more oxygen vacancies, and more positive surface potential exhibit a higher ammonia yield rate of 349 ± 15 μg h^–1^ mg^–1^_cat._ and a Faradaic efficiency of 48 ± 2% than LF submicrofibers. The *COMSOL* Multiphysics simulations demonstrate that the more positive surface of LF_0.9_Cu_0.1_ submicrofibers can induce NO_3_^–^ enrichment and suppress the competing hydrogen evolution reaction. By combining a variety of *in situ* characterizations and density functional theory calculations, the eNITRR mechanism is revealed, where the first proton–electron coupling step (*NO_3_ + H^+^ + e^–^ → *HNO_3_) is the rate-determining step with a reduced energy barrier of 1.83 eV. This work highlights the positive effect of cation substitution in promoting eNITRR properties of perovskites and provides new insights into the studies of perovskite-type electrocatalytic ammonia synthesis catalysts.

## 1 Introduction

Ammonia (NH_3_), an essential chemical in the nitrogen cycle network, is a fundamental compound for human beings that has been widely used as a nitrogen source for the synthesis of fertilizers, explosives, plastics, and so on.^1^ Currently, NH_3_ is mainly produced *via* the energy-intensive Haber–Bosch process under harsh conditions (300–500 °C, 200–300 bar),^2^ accompanied by an abundant amount of greenhouse gas emissions (the global average is 2.86 tons of carbon dioxide per ton of NH_3_).^3^ Thus, alternative approaches such as the electrocatalytic nitrogen reduction reaction (eNRR) under mild operating conditions have become more popular in recent years.^4–8^ Recent research indicates that metals, alloys, nitrides, oxides, and carbides can function as potential eNRR catalysts.^9^ Despite significant achievements in recent years, the eNRR still suffers from low Faradaic efficiencies (FEs, mostly <30%) and limited NH_3_ yield rates (mostly <200 μg h^–1^ mg^–1^_cat._).^9,10^ These challenges arise from the extremely stable N≡N triple bond with a bond energy of 941 kJ mol^–1^, the low solubility of N_2_ gas in aqueous electrolytes, and the competing hydrogen evolution reaction (HER).^11–13^ Owning to the high solubility (the solubility of NaNO_3_ is 87.6 g in 100 mL of water at 293 K) and the weak N=O double bond (binding energy of 204 kJ mol^–1^) of nitrate (NO_3_^–^), the electrocatalytic nitrate reduction reaction (eNITRR) has emerged as an alternative technology to the eNRR process that can improve both the NH_3_ yield rate and the FE.^14,15^ The NO_3_^–^ ion is a pollutant found in many types of wastewaters.^16–19^ Therefore, finding ways to use it by the eNITRR can also tackle environmental issues simultaneously. However, designing advanced electrocatalysts with high selectivity for transforming NO_3_^–^ to NH_3_*via* the eNITRR remains a challenge because the eNITRR is a complicated 8e^–^ transfer reaction involving many intermediates (*e.g.*, NO_2_, NO, N_2_O, N_2_, NH_2_OH, NH_3_, and NH_2_NH_2_).^15^ Among the various eNITRR electrocatalysts, Cu-containing catalysts demonstrate superior performance compared to other catalysts.^20^ However, the issue of instability in copper-containing catalysts limits their applications, which has also sparked research interest in the reaction mechanism of Cu-containing catalysts and the design of highly stable Cu-containing catalysts. For instance, extensive research has been conducted on various aspects, such as regulating the d-band center of Cu-containing catalysts,^14^ the electrochemical restructuring of Cu-containing catalysts,^20–22^ and the synergistic effects between Cu and other metals.^23,24^ Nonetheless, addressing the stability concern associated with Cu still poses a significant challenge.

The previous search for cost-effective and efficient eNITRR catalysts has revealed the potential of perovskite oxides owing to their flexible electronic structures and chemical versatility.^25–27^ Perovskite oxides (ABO_3_, where the A-site cations are alkaline-earth or rare-earth metals and B-site cations are transition metals) are conventionally prepared by ball milling or sol–gel methods, resulting in an uncontrollable and blocked structure with severe particle agglomeration.^28,29^ Generally, it has been demonstrated that exposing accessible active sites of perovskite oxides by constructing specific geometrical structures, such as SrNb_0.1_Co_0.7_Fe_0.2_O_3−δ_ nanorods,^30^ BiFeO_3_ nanosheets,^31^ NaNbO_3_ nanocubes,^32^ and so on, can result in a highly efficient electrocatalytic process. Among them, the cross-linked network woven by one-dimensional (1D) perovskite oxide submicrofibers can not only facilitate reactant diffusion and electron transport during the electrocatalytic process but also avoid particle agglomeration to expose the maximum number of active sites for the adsorption and reduction of NO_3_^–^ ions. In addition to the construction of the unique structure, the activity of the perovskite is also affected by the electronic environment around the active sites.^33^ As the perovskite oxides can accommodate ∼90% of metallic elements of the periodic table, their high compositional flexibility enables the incorporation of metal elements to form a B-site bimetallic perovskite. Based on the molecular orbital theory and band theory, the catalytic performance of perovskite oxides can be influenced by tailoring the octahedral structure of their [BO_6_] units, regulating the hybridization of B–O bonds, and generating oxygen vacancies (OVs). For example, the covalency of transition metal–oxygen bonds, which reflects the adsorption strength of oxygen-related intermediates, was verified to be an important descriptor for electrocatalytic activity.^28,34^ Therefore, it is rational to optimize the adsorption strength of intermediates in the eNITRR process by substituting the B-site cation with other elements. Apart from the variation of the electronic structure, the B-site cation substitution strategy will also affect the work function of the catalyst surface, which is accompanied by a change in surface potential.^35,36^ The surface potential and corresponding work function play important roles in facilitating the transport of electrons from the catalyst to the reactant.

Inspired by the Fe active sites in both Haber–Bosch catalysts (Fe-based compounds) and nitrogenase enzymes (mainly containing the Fe–Mo cofactor), a series of Fe-rich perovskite oxides of LaFe_0.9_M_0.1_O_3−δ_ (M = Co, Ni, and Cu) submicrofibers (noted as LF_0.9_Co_0.1_, LF_0.9_Ni_0.1_, and LF_0.9_Cu_0.1_ submicrofibers, respectively) were constructed by a B-site substitution strategy and acted as the eNITRR electrocatalysts. The LF_0.9_Cu_0.1_ submicrofibers showed a higher NH_3_ yield rate of 349 ± 15 μg h^–1^ mg^–1^_cat._ and a higher FE of 48 ± 2% than the parent LF submicrofibers, which are attributed to the inhomogeneous charge redistribution and abundant OVs on the surface of LF_0.9_Cu_0.1_ submicrofibers, as well as its more positive surface potential and lower work function for achieving an enhanced adsorption ability toward NO_3_^–^ ions. *COMSOL* Multiphysics simulations also confirm this discovery from the perspective of a theoretical calculation. The reaction mechanism was investigated in detail by combining operando Fourier transform infrared (FT-IR) spectroscopy, online differential electrochemical mass spectrometry (DEMS), and density functional theory (DFT) calculations. In the deoxidation process of the reaction stages, the first proton–electron coupling step of *NO_3_ + H^+^ + e^–^ → *HNO_3_ is the rate-determining step (RDS). The substitution of Cu in the B site lowers the barrier energy in this step, thereby facilitating the reaction.

## 2 Results and Discussion

**Figure 1:**
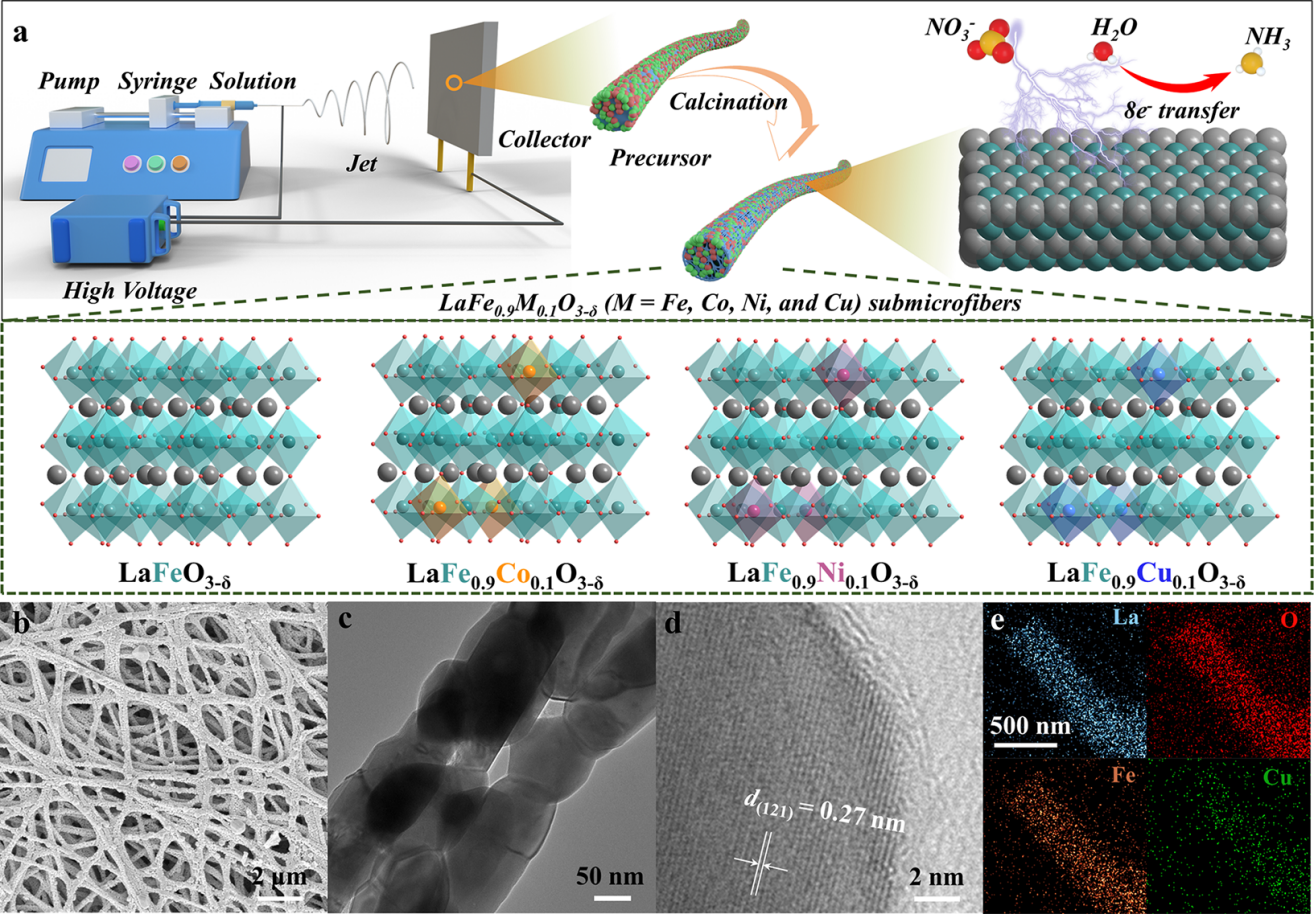
(a) Schematic illustration for the synthetic process of 1D perovskite submicrofibers. (b) SEM image, (c) TEM image, (d) HRTEM image, and (e) EDX mappings of LF_0.9_Cu_0.1_ submicrofibers.

The LaFeO_3_ (LF) and LF_0.9_M_0.1_ (M = Co, Ni, and Cu) submicrofibers were synthesized *via* an electrospinning technique followed by calcination, as illustrated in Figure 1a. In a typical procedure, the metal precursors as nitrate salts and polyvinylpyrrolidone were dissolved in *N*,*N*-dimethylformamide to form a homogeneous, viscous solution, which was used for the electrospinning process. Subsequently, the electrospun metal salt–polymer submicrofibers were calcined to generate 1D perovskite oxide submicrofibers (more experimental details can be found in the Supporting Information). As the scanning electron microscopy (SEM) images show in Figures 1b and S1, all these samples consist of 1D submicrofibers without obvious fracture or aggregation. As an example, LF_0.9_Cu_0.1_ submicrofibers are stacked layer-by-layer and connected to form three-dimensional networks (Figure 1b), with an average diameter of approximately 250 nm (Figure S2). Notably, the LF_0.9_Cu_0.1_ submicrofiber was generated by concatenating abundant LF_0.9_Cu_0.1_ nanoparticles, with diameters ranging between 50 and 70 nm, into an integrated structure. This is evident from the transmission electron microscopy (TEM) image (Figure 1c). The high-resolution TEM (HRTEM) image of LF_0.9_Cu_0.1_ shows clear crystal fringes with a lattice spacing of ∼0.27 nm, which belongs to the (121) plane of the perovskite oxide (Figure 1d). Moreover, the energy-dispersive X-ray spectroscopy (EDX) mappings of LF_0.9_Cu_0.1_ submicrofibers (Figure 1e) show that La, Fe, O, and Cu elements are dispersed throughout the nanostructure homogeneously. The perovskite crystal phases of LF, LF_0.9_Co_0.1_, LF_0.9_Ni_0.1_, and LF_0.9_Cu_0.1_ submicrofibers were verified by X-ray diffraction (XRD) patterns, as shown in Figure S3. All samples display clear diffraction peaks at 22.6, 25.3, 32.2, 39.7, 46.1, 47.6, 52.0, 53.3, 57.4, 67.3, and 76.7°, which can be indexed to the (101), (111), (121), (220), (202), (212), (103), (311), (123), (242), and (204) planes, respectively, of orthorhombic perovskite structures (JCPDS 88–0641) without any impurity phase. It should be noted that the diffraction peaks would shift slightly after cationic substitution, such as the (121) peak, which may imply the lattice variation due to the cation substitution.^37^ The crystal structures were further confirmed by Rietveld refinement of the XRD patterns (Figure S4) in Table S1. By taking LF_0.9_Cu_0.1_ as an example, the decrease in unit cell volume indicates that the substitution of Cu caused lattice shrinkage due to the reduced ionic radii of Cu^2+^ ions. The atomic ratios of Fe/M in LF_0.9_Co_0.1_, LF_0.9_Ni_0.1_, and LF_0.9_Cu_0.1_ submicrofibers were also checked to be approximately 9:1 by inductively coupled plasma mass spectrometry (ICP–MS) (Table S2). In short, the 1D perovskite submicrofibers formed by the directional accumulation of nanoparticles with tunable B-site bimetallic cations were successfully synthesized by the electrospinning technique and calcination process.

**Figure 2:**
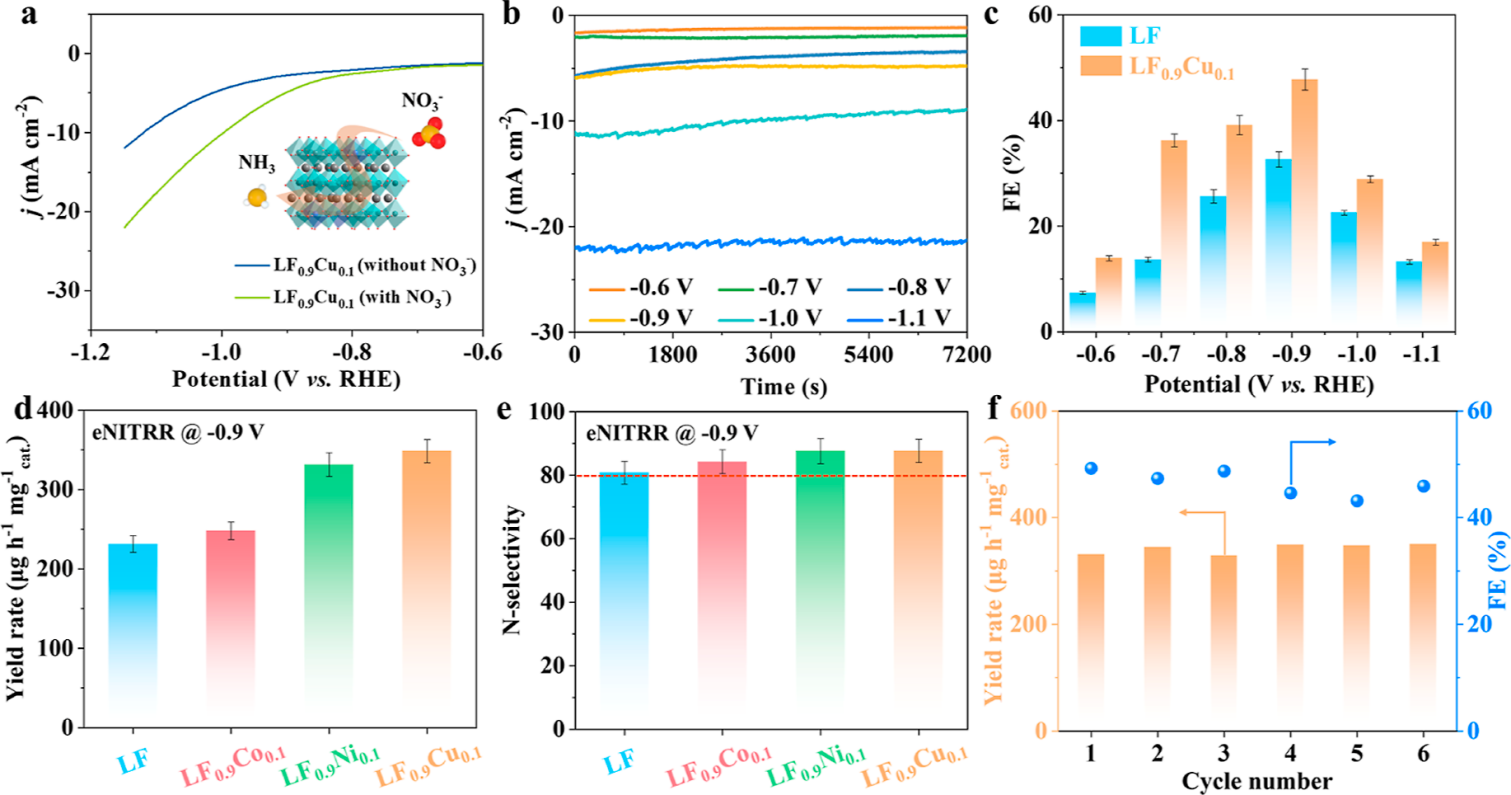
(a) LSV curves of LF_0.9_Cu_0.1_ submicrofibers in 0.5 M Na_2_SO_4_ electrolyte with and without NO_3_^–^. (b) Time-dependent current density curves of the LF_0.9_Cu_0.1_ submicrofibers against various work potentials. (c) FE values of LF and LF_0.9_Cu_0.1_ submicrofibers at each given potential. (d) NH_3_ yield rates and (e) N selectivities of LF and LF_0.9_M_0.1_ (M = Co, Ni, and Cu) submicrofibers at −0.9 V *vs* RHE. (f) Cycling tests of LF_0.9_Cu_0.1_ submicrofibers at −0.9 V *vs* RHE.

The eNITRR performance of various perovskite submicrofibers was evaluated in an H-type electrolytic cell under ambient conditions, where 0.5 M Na_2_SO_4_ with 50 ppm NO_3_^–^–N (NO_3_^–^–N represents the concentration of NO_3_^–^ expressed in terms of the nitrogen content) aqueous solution was used as the electrolyte. The concentrations of reactant NO_3_^–^, reductive product NH_3_, and byproduct nitrite (NO_2_^–^) were detected using colorimetric methods (Figures S5–S7).^38–40^ The linear sweep voltammetry (LSV) curves of LF_0.9_Cu_0.1_ submicrofibers in 0.5 M Na_2_SO_4_ electrolytes with and without NO_3_^–^ were recorded to investigate the potential window for the eNITRR process. When the potential is more negative than −0.6 V *vs* the reversible hydrogen electrode (RHE), the current density for the LSV curve with NO_3_^–^ is enhanced significantly (Figure 2a), suggesting the occurrence of the eNITRR process. Figure 2b shows the time-dependent current density curves of LF_0.9_Cu_0.1_ submicrofibers at different working potentials. The negligible decay in current density indicates the considerable catalytic stability of LF_0.9_Cu_0.1_ submicrofibers. From the summarized FE values and NH_3_ yield rates for various samples (LF, LF_0.9_Co_0.1_, LF_0.9_Ni_0.1_, and LF_0.9_Cu_0.1_) in Figures 2c,d, S8, and S9, the FEs for NH_3_ of these samples follow a volcano-type trend as a function of applied potentials. This phenomenon can be attributed to the increased competition from the HER at more negative potentials, coupled with a decrease in the available charges at more positive potentials. Notably, the LF_0.9_Cu_0.1_ submicrofibers exhibit the best eNITRR performance with a maximal FE value of 48 ± 2% and a high NH_3_ yield rate of 349 ± 15 μg h^–1^ mg^–1^_cat._ at −0.90 V *vs* RHE. Additionally, the ^1^H nuclear magnetic resonance method (Figure S10) was also conducted as another quantitative measurement to check the NH_3_ yield rates for all electrocatalysts at a potential of −0.9 V *vs* RHE, which are in good agreement with the results by the UV–vis spectroscopy method (Figure S11). The N-selectivity indicates the ratio of NH_4_^+^–N in converted NO_3_^–^–N, and is an important parameter that reflects the degree of conversion from NO_3_^–^ to NH_4_^+^. By testing the NO_3_^–^ concentrations for various samples after 2 h eNITRR processes at −0.9 V *vs* RHE, it can be observed that all the perovskite oxides display high N-selectivities of NH_3_ of over 80% (Figure 2e), which are comparable to or better than other reports for NO_3_^–^ electroreduction (Table S3). The N-selectivities of the four samples for NO_2_^–^ are close to 20%, indicating that their main soluble byproduct is NO_2_^–^ (Figure S12). To evaluate the eNITRR performance of LF_0.9_Cu_0.1_ comprehensively, the NH_3_ yield rates and FEs at different concentrations were also measured. The results, as depicted in Figure S13, demonstrate that the catalyst maintains high catalytic activity even at higher concentrations.

To exclude the interference from the electrocatalyst itself or the external environment, a comparison test was performed in 0.5 M Na_2_SO_4_ electrolyte without NO_3_^–^. As shown in Figure S14, no NH_3_ can be detected, which indicates that the produced NH_3_ during the eNITRR process originates from the NO_3_^–^ ions in the Na_2_SO_4_ electrolyte. Furthermore, the electrocatalytic and structural stabilities of the LF_0.9_Cu_0.1_ submicrofibers were evaluated by 6 consecutive electrolysis cycles at −0.9 V *vs* RHE. As shown in Figure 2f, the NH_3_ yield rate and FE value of each cycle fluctuate slightly but remain stable, suggesting the excellent electrocatalytic stability of LF_0.9_Cu_0.1_ submicrofibers for potential practical applications. The perovskite structure of LF_0.9_Cu_0.1_ submicrofibers is also maintained well based on the XRD pattern of LF_0.9_Cu_0.1_/carbon paper after the eNITRR process (Figure S15), which illustrates the excellent structural stability of LF_0.9_Cu_0.1_ submicrofibers. ICP–MS was employed for the evaluation of changes in the composition of LF_0.9_Cu_0.1_ submicrofibers after the stability test. The results show that no significant changes have occurred in the composition of the catalyst, further highlighting its excellent stability (Table S4). Figure S16 shows the Nyquist plots of the LF and LF_0.9_Cu_0.1_ submicrofibers. The charge transfer resistance of LF_0.9_Cu_0.1_ (28.6 ± 3.9 Ω) is smaller than that of LF (40.0 ± 3.0 Ω), which indicates that the electron transfer rate is faster in LF_0.9_Cu_0.1_. To investigate the influence of active sites on the eNITRR, we calculated the electrochemically active surface areas (ECSAs) of LF and LF_0.9_Cu_0.1_. As shown in Figures S17 and S18, the double-layer capacitance of LF_0.9_Cu_0.1_ is 0.31 mF cm^–2^, which is almost equal to that of LF at 0.27 mF cm^–2^. Based on this, the activity gap between LF and LF_0.9_Cu_0.1_ was not strongly correlated with the ECSA.

**Figure 3:**
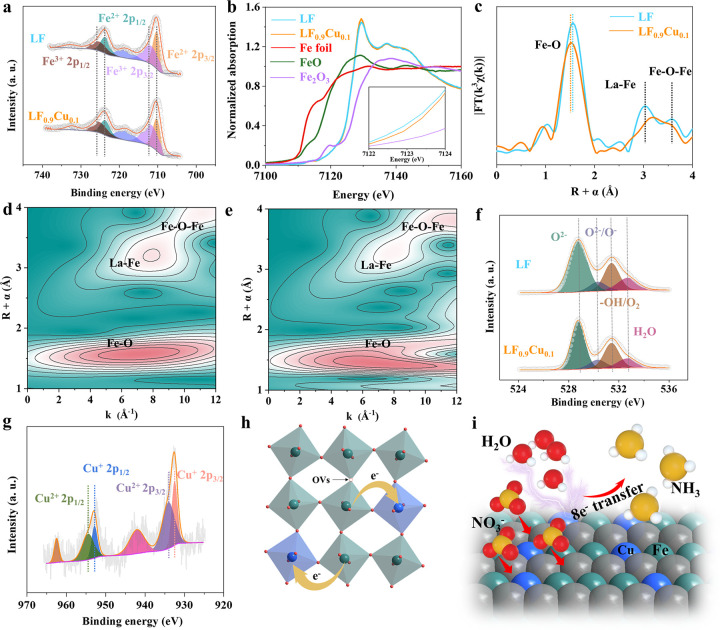
(a) Fe 2p XPS spectra in LF and LF_0.9_Cu_0.1_ submicrofibers. (b) Fe K-edge XANES spectra (inset: enlarged view of the absorption edge position) of LF, LF_0.9_Cu_0.1_, Fe foil, FeO, and Fe_2_O_3_. (c) Fe K-edge FT-EXAFS spectra of LF and LF_0.9_Cu_0.1_. WT-EXAFS at Fe K-edge of (d) LF and (e) LF_0.9_Cu_0.1_. (f) O 1s XPS spectra of LF and LF_0.9_Cu_0.1_ submicrofibers. (g) Cu 2p XPS spectrum of LF_0.9_Cu_0.1_ submicrofibers. (h) Schematic illustration of the charge redistribution and generation of OVs in LF_0.9_Cu_0.1_. (i) Illustration for the eNITRR on the surface of LF_0.9_Cu_0.1_ submicrofibers.

The valence state and electronic structural information were explored by X-ray photoelectron spectroscopy (XPS) and synchrotron-based X-ray absorption spectroscopy (XAS) techniques. Figure 3a shows Fe 2p XPS spectra of LF and LF_0.9_Cu_0.1_ submicrofibers, and the peaks at 710.2 eV (Fe^2+^ 2p_3/2_), 712.1 eV (Fe^3+^ 2p_3/2_), 723.7 eV (Fe^2+^ 2p_1/2_), and 725.5 eV (Fe^3+^ 2p_1/2_) indicate the coexistence of Fe^2+^ and Fe^3+^.^41,42^ The Fe^3+^/Fe^2+^ ratio increases with the substitution of low-valent Cu atoms in LF because more Fe^3+^ ions are required to balance the charges (Table S5).^43^ This phenomenon also illustrates the charge redistribution in LF as a result of the cation substitution strategy.^44^ Moreover, the valence state of Fe is influenced by the filling degree of the d orbitals, thereby determining the d-band center of the catalyst. Therefore, the value for the d-band center of the Fe element becomes more positive with the increase of its valence state, leading to an enhanced adsorption of NO_3_^–^ ions.^45^ The Fe coordination environments of LF and LF_0.9_Cu_0.1_ were also investigated by synchrotron-based XAS measurements. The X-ray absorption near-edge structure (XANES) spectra of LF, LF_0.9_Cu_0.1_, standard Fe_2_O_3_, standard FeO, and Fe foil are shown in Figure 3b. The Fe K-edge XANES spectra of LF and LF_0.9_Cu_0.1_ are found to be between those of FeO and Fe_2_O_3_, suggesting that their average valence states of Fe are both between +2 and +3. Meanwhile, XANES spectra of Fe in LF_0.9_Cu_0.1_ has a higher energy than that in LF, indicating that the valence state of Fe in LF_0.9_Cu_0.1_ is slightly higher than that in LF,^46^ which is consistent with the XPS results. The Fourier transform extended X-ray absorption fine structure (FT-EXAFS) spectra in *R*-space for LF and LF_0.9_Cu_0.1_ are shown in Figure 3c. There are three prominent peaks at about 1.5, 3.0, and 3.5 Å for LF and LF_0.9_Cu_0.1_ that originate from the scattering paths of the Fe–O, La–Fe, and Fe–O–Fe bonds,^47^ respectively. With partial Fe atoms substituted by Cu atoms, the length of the Fe–O bond decreases and indicates an enhanced hybridization of the Fe 3d–O 2p orbitals.^48^ The wavelet transform (WT)-EXAFS measurements were also used to provide insights into the coordination structures of Fe atoms in LF and LF_0.9_Cu_0.1_ (Figures 3d,e and S19). The maximum intensity is closely associated with the path length, which can provide pivotal clues for identifying the coordination environment. Specifically, the scattering path signals at [χ(*R*), χ(*k*)] of [1.56, 7.0] and [1.50, 6.9] are associated with Fe–O bonds in the LF and LF_0.9_Cu_0.1_ submicrofibers, respectively. The second set of WT-EXAFS peaks at [3.0, 7.9] and [3.0, 7.8] can be assigned to the La–Fe contributions in the LF and LF_0.9_Cu_0.1_ submicrofibers, respectively. The third set of WT-EXAFS peaks at [3.5, 9.3] and [3.5, 8.9] originate from the Fe–O–Fe bonds in the LF and LF_0.9_Cu_0.1_ submicrofibers, respectively. It is further demonstrated that the bond length of Fe–O reduces after Cu substitution, which agrees with the results of the FT-EXAFS spectra in *R* space. The fitting curves at the *R* space and *k* space are consistent with the FT-EXAFS spectra of LF and LF_0.9_Cu_0.1_ (Figures S20 and S21). Based on the above analysis, it is evidenced that the B-site substitution strategy with low-valent metal elements can lead to charge redistribution and curtate Fe–O bond distance.^49^ It can be further inferred that with the increase of Fe 3d–O 2p orbital hybridization, the lattice O p bands shift toward the *E*_Fermi_ as the Fe d states shift closer to the lattice O p energy.^35,48^ The shift of the lattice O p band lowers the formation energy of OVs. Subsequently, it facilitates the generation of more OVs on the surface, promoting full contact between the reactant and active B-site transition metals. The OVs in LF and LF_0.9_Cu_0.1_ were probed *via* the XPS spectra of O 1s species and electron paramagnetic resonance (EPR) spectra. The corresponding deconvolution results of O 1s are shown in Figure 3f. The peaks at 528.8, 530.4, 531.4, and 532.7 eV are assigned to lattice O^2–^, a highly oxidative oxygen species (O_2_^2–^/O^–^), surface-adsorbed O_2_ or hydroxyl groups, and surface-adsorbed H_2_O, respectively.^38,50^ Based on the relative areas of peaks (Table S6), the concentrations of surface OVs, which are correlated with the O_2_^2–^/O^–^ species,^51^ were calculated to be 13.6 and 17.6% for LF and LF_0.9_Cu_0.1_, respectively. These results indicate that the surface OVs slightly increased with Cu substitution. To further study the variation of OVs, the EPR spectra of LF and LF_0.9_Cu_0.1_ submicrofibers were also recorded, as shown in Figure S22. Both samples exhibit EPR signals at *g* = 2.004, which are identified as the trapped electrons in the OVs.^52^ Furthermore, the EPR signal intensity of LF_0.9_Cu_0.1_ is higher than that of LF, indicating that more OVs have been generated in LF_0.9_Cu_0.1_. The chemical state of Cu of the LF_0.9_Cu_0.1_ submicrofibers was subsequently checked by XPS measurements (Figure 3g). The four peaks at 932.4 eV (Cu^+^ 2p_3/2_), 934.0 eV (Cu^2+^ 2p_3/2_), 952.8 (Cu^+^ 2p_1/2_), and 954.5 eV (Cu^2+^ 2p_1/2_) suggest that there are two chemical states for the Cu element in LF_0.9_Cu_0.1_ submicrofibers (Table S7).^53^ Considering the potential stability issues associated with Cu during the eNITRR process, XPS measurements of Cu were also conducted after the stability test for LF_0.9_Cu_0.1_ (Figure S23). The results demonstrate that except for a slight decrease in the oxidation state, Cu still maintains the same two chemical states (Table S8). Following the bespoke analysis, it can be concluded that the charge redistribution and increased surface OVs induced by the B-site substitution strategy are expected to promote the eNITRR catalytic activity (Figure 3h–i).

**Figure 4:**
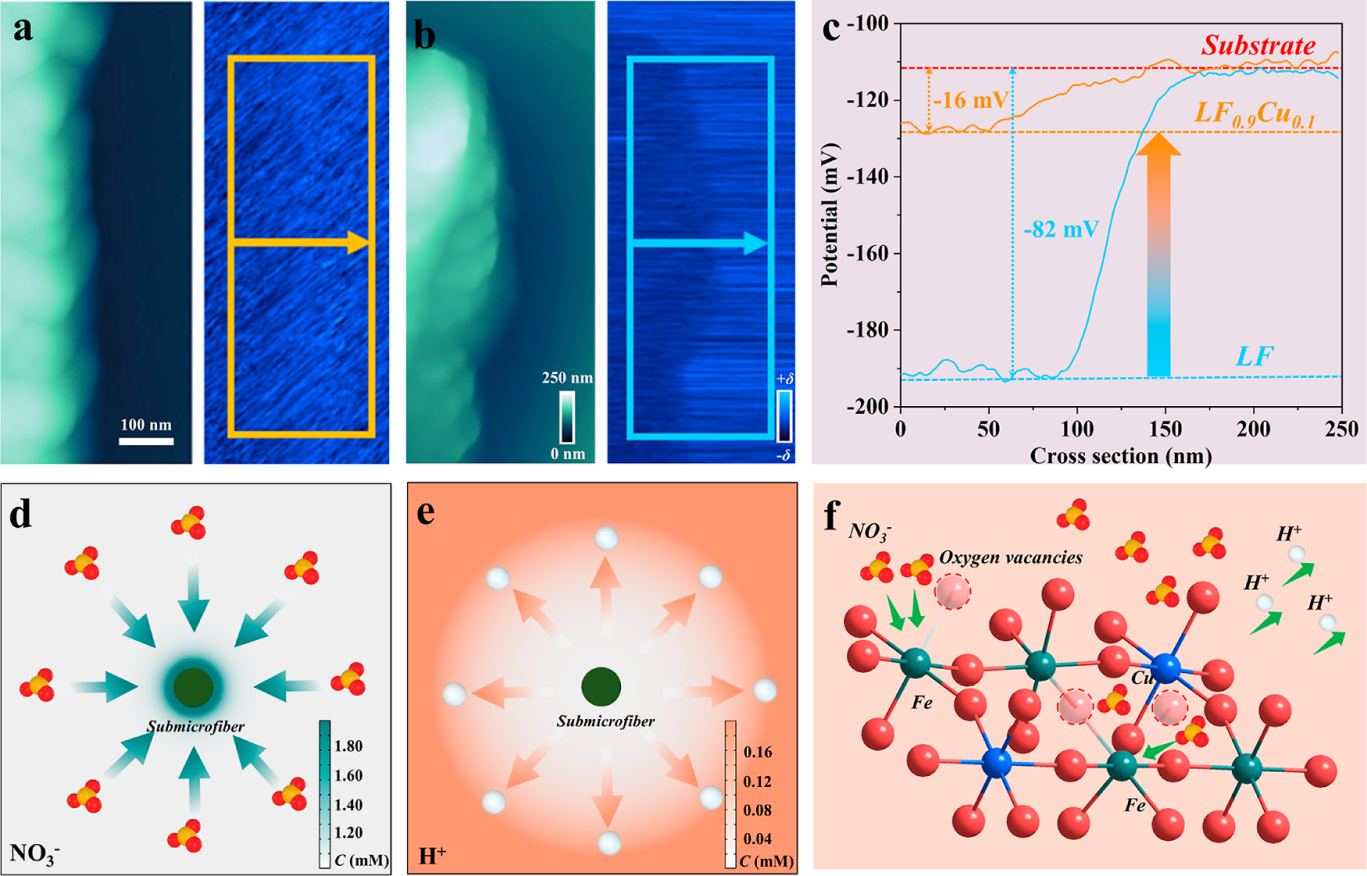
Surface potential distribution of (a) LF_0.9_Cu_0.1_ and (b) LF submicrofibers. (c) Surface potential values extracted in (a,b). Top view of the model of the variation of (d) NO_3_^–^ and (e) H^+^ concentrations on the fiber surface. (f) Schematic illustration of the ion movement on the catalyst surface with more positive potential.

The surface potential will be changed by B-site substitution in the perovskite structure.^35,36^ Therefore, Kelvin probe force microscopy (KPFM) was conducted to investigate the surface potential of the LF and LF_0.9_Cu_0.1_ submicrofibers. The potential distributions at the LF_0.9_Cu_0.1_ and LF submicrofiber surfaces are shown in Figure 4a,b with their corresponding topography images. A rectangular region (along the direction of the arrow) was chosen to quantify the surface potential variation. As shown in Figure 4c, the surface potential of LF_0.9_Cu_0.1_ is roughly 66 mV higher than that of LF. The above results show that the work function of LF_0.9_Cu_0.1_ is about 0.066 eV lower than that of LF according to the conversion formula between the work function and surface potential.^36^ A lower work function of LF_0.9_Cu_0.1_ submicrofibers implies a smaller energy barrier for electron transfer to the reactant (NO_3_^–^).^54^ Notably, the surface potential of LF_0.9_Cu_0.1_ submicrofibers becomes more positive, which indicates that LF_0.9_Cu_0.1_ is more favorable to adsorbing and enriching NO_3_^–^. Therefore, it is advantageous by constructing eNITRR catalysts with more positive surface potential in addressing the issue of low activity resulting from weak mass transfer and small concentration gradients near the electrode region in low-concentration NO_3_^–^ electrolytes.^55^ Meanwhile, *COMSOL* Multiphysics simulations were used to simulate the ionic distribution after the variation of the potential on the nanoreactor surface. Since the competitive HER can inhibit the eNITRR process, the concentration of hydrogen ions on the catalyst surface is also an important factor affecting the eNITRR performance. The variations of ion concentrations are described through the Nernst–Planck equation (see the details in the Supporting Information). Before the simulation, it is assumed that the anions and cations are distributed randomly, without electrostatic force. After the surface potential becomes more positive, the distributions of the NO_3_^–^ and H^+^ ions are shown in Figures 4d,e and S24. The NO_3_^–^ concentration increases gradually, indicating a NO_3_^–^ enrichment. On the contrary, the H^+^ concentration at the surface of LF_0.9_Cu_0.1_ submicrofibers decreases progressively. These results demonstrate that a surface with more positive potential would be beneficial for not only promoting the enrichment of NO_3_^–^ ions but also reducing the HER process (Figure 4f).

**Figure 5:**
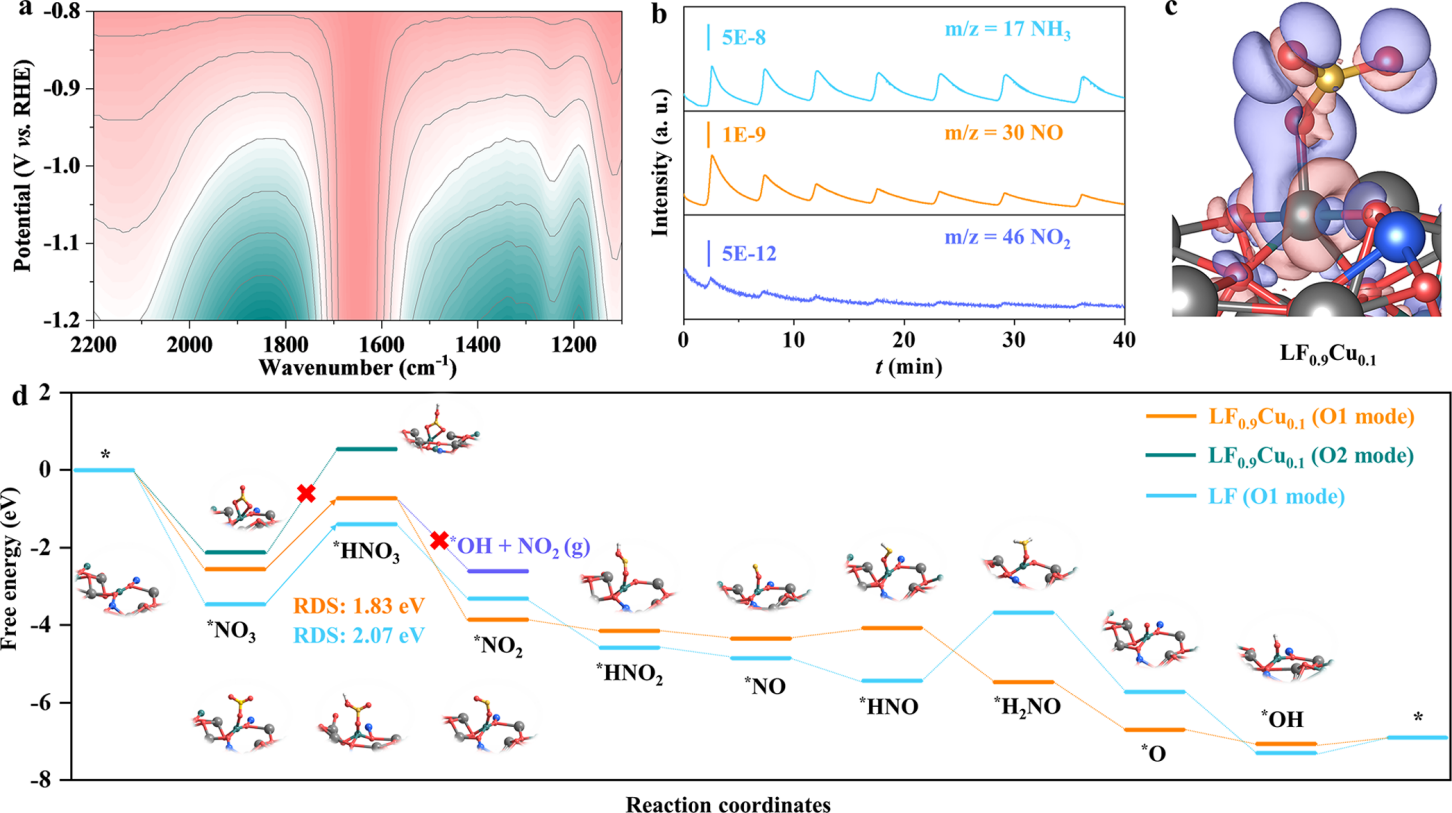
(a) Two-dimensional operando FT-IR spectra of LF_0.9_Cu_0.1_. Here, the pink represents high values of transmittance, while the green represents low values of transmittance. (b) DEMS signals of gaseous intermediates/products during seven cycles. (c) Charge density difference of LF_0.9_Cu_0.1_ coupled with NO_3_^-^. (d) Gibbs free energy diagrams of the eNITRR on the surface of LF and LF_0.9_Cu_0.1_.

The mechanism for the eNITRR process was experimentally probed by two-dimensional operando FT-IR spectroscopy and online DEMS. The infrared signals are collected from 1100 to 2200 cm^–1^ during a negative scan from −0.8 to −1.2 V *vs* RHE (Figure 5a). It can be concluded that as the potentials become more negative, the intensity of the characteristic peaks increases. This demonstrates the emergence of the rocking mode of –NH_2_ at 1190 cm^–1^,^56^ the wagging mode of –NH_2_ at 1307 cm^–1^,^57^ and M–N–O groups in the range of 1800–2000 cm^–1^.^58^ Moreover, the molecular intermediates and products were detected by online DEMS with an applied voltage between −0.6 and −1.2 V *vs* RHE (Figure 5b). The *m*/*z* signals of 46, 30, and 17 appear during seven subsequent cycles and correspond to the NO_2_, NO, and NH_3_ species, respectively.

DFT calculations were also conducted to explore the relationship between the Cu substitution and improved eNITRR activity. The charge density difference of LF_0.9_Cu_0.1_ after adsorbing NO_3_^–^ is plotted in Figure 5c, which shows a charge transfer from Fe to adsorbed NO_3_^–^ and provides clues into the adsorption and activation of NO_3_^–^ for subsequent hydrogenation. Based on the findings from DEMS/FT-IR spectroscopy measurements, the reaction pathway of NO_3_^–^ electroreduction could be deduced, and the free energy of every intermediate over LF and LF_0.9_Cu_0.1_ was calculated. According to a previous study,^38,59^ the eNITRR process can be considered as a hydrogenation process (Figure S25). Before the calculation of the total reaction steps, it is crucial to determine the mode of NO_3_^–^ adsorption and hydrogenation on the LF_0.9_Cu_0.1_ surface. The free energies of two different pathways to form *NO_3_ and *HNO_3_ were calculated. As shown in Figure 5d, the free energy values of the NO_3_^–^ coupling and first proton–electron coupling steps of single oxygen (O1 mode) on the Fe site are lower than those of dioxygen (O2 mode). For example, the free energy values for the first proton–electron coupling step (*NO_3_ + H^+^ + e^–^ → *HNO_3_) with the O1 and O2 modes are 1.83 and 2.67 eV, respectively, which indicate that the O1 mode is a more optimal structure for the coupling step between the LF_0.9_Cu_0.1_ catalyst and NO_3_^–^ species, as well as the following hydrogenation. It is noted that the byproduct NO_2_(g) can be produced in the side reaction *HNO_3_ + H^+^ + e^–^ → *OH + NO_2_(g). The free energy of this potential intermediate was also calculated with a larger free energy of −2.61 eV than that of the step for the generation of the *NO_2_ species (−3.86 eV). Therefore, the side reaction is much less likely to occur than the second proton–electron coupling step (*HNO_3_ + H^+^ + e^–^ → H_2_O + *NO_2_). After other possibilities are excluded, the total reaction pathway is shown as follows. First, the reactant NO_3_^–^ is chemically absorbed on the catalyst surface to form *NO_3_ with a decrease of total energy, indicating the driving force of this reaction step. Then, the N–O bond is continuously cleaved by proton-coupled electron transfer to form *NO_2_ and *NO. During the following hydrogenation processes, the *NO intermediate is converted to *HNO, *H_2_NO, and *O species gradually. In the final active site refreshing process, the *O intermediate is coupled with two other protons and converted to a H_2_O molecule. To be noted, different intermediates were adsorbed on the surface of catalysts, and the most stable adsorption models are employed to describe the eNITRR process (illustrations of Figure 5d). For both LF_0.9_Cu_0.1_ and LF surfaces, nonspontaneous reaction steps can be clearly observed that will influence the reaction rate of the eNITRR process. Among these nonspontaneous steps, the RDS is the first proton–electron coupling step (*NO_3_ + H^+^ + e^–^ → *HNO_3_), where the energy barriers are 2.07 and 1.83 eV for LF and LF_0.9_Cu_0.1_, respectively. This indicates that the eNITRR process can be facilitated on the surface of LF_0.9_Cu_0.1_. Combining all experimental results with the theoretical calculations, it can be concluded that the eNITRR performance can be promoted by the B-site substitution strategy.

## 3 Conclusions

In summary, we have demonstrated that perovskite LF_0.9_Cu_0.1_ submicrofibers can act as a selective and efficient electrocatalyst to reduce NO_3_^–^ to valuable NH_3_. At the optimal potential of −0.9 V, the NH_3_ yield rate, FE, and selectivity can reach 349 ± 15 μg h^–1^ mg^–1^_cat._, 48 ± 2%, and 88 ± 4%, respectively. Synchrotron-based XAS revealed the charge redistribution on the L_0.9_Cu_0.1_ submicrofiber and confirmed the increase of hybridization of the Fe 3d–O 2p orbital, which favors the generation of surface OVs. Combined with the results of KPFM and *COMSOL* Multiphysics simulations, it is shown that a more positive surface can be induced by the cation substitution strategy, which promotes the fixation of more NO_3_^–^ anions on the catalyst surface. Based on a variety of *in situ* characterizations and DFT calculations, the reaction pathway was deduced. The results showed that the lower free energy for the RDS (*NO_3_ + H^+^ + e^–^ → *HNO_3_) for LF_0.9_Cu_0.1_ led to its optimal NH_3_ synthesis performance. This work opens new avenues for designing efficient perovskite-type electrocatalytic NH_3_ synthesis catalysts *via* a cation substitution strategy.

## Supporting Information Available

Detailed experimental procedures, supported physical and electrochemical characterization of materials by SEM, TEM, XRD, ^1^H nuclear magnetic resonance, XPS, cyclic voltammetry, EPR, ICP-MS, and additional EXAFS results (PDF)
