# Supplementary material for: Cation Substitution Strategy for Developing Perovskite Oxide with Rich Oxygen Vacancy-Mediated Charge Redistribution Enables Highly Efficient Nitrate Electroreduction to Ammonia
Source: J Am Chem Soc. 2023 Sep 20;145(39):21387–96. doi: 10.1021/jacs.3c06402 (PMC10557098; doi:10.1021/jacs.3c06402)
Supplement: Supplementary file 1 — ja3c06402_si_001.pdf [file ja3c06402_si_001.pdf]

Supporting Information

# Cation Substitution Strategy for Developing Perovskite Oxide with Rich Oxygen Vacancy-Mediated Charge Redistribution Enables Highly Efficient Nitrate Electroreduction to Ammonia

Kaibin Chu[1,2], Wei Zong[1], Guohao Xue[1], Hele Guo[2], Jingjing Qin[1], Haiyan Zhu[1], Nan Zhang[1], Zhihong Tian[3], Hongliang Dong[4], Yue-E. Miao[5], Maarten B. J. Roeffaers[6], Johan Hofkens[2,7], Feili Lai[2,7,8*], Tianxi Liu[1*]

[1] The Key Laboratory of Synthetic and Biological Colloids, Ministry of Education, School of Chemical and Material Engineering, International Joint Research Laboratory for Nano Energy Composites, Jiangnan University, Wuxi 214122, China

[2] Department of Chemistry, KU Leuven, Celestijnenlaan 200F, Leuven 3001, Belgium

[3] Engineering Research Center for Nanomaterials, Henan University, Kaifeng 475004, China

[4] Center for High Pressure Science and Technology Advanced Research, Shanghai 201203, China

[5] State Key Laboratory for Modification of Chemical Fibers and Polymer Materials, College of Materials Science and Engineering, Donghua University, Shanghai 201620, China

[6] cMACS, Department of Microbial and Molecular Systems, KU Leuven, Celestijnenlaan 200F, Leuven 3001, Belgium

[7] Max Planck Institute for Polymer Research, Ackermannweg 10, Mainz 55128, Germany

[8] John A. Paulson School of Engineering and Applied Sciences, Harvard University, Cambridge, Massachusetts 02138, United States

[*] Corresponding authors (emails: feili.lai@kuleuven.be; txliu@jiangnan.edu.cn)

## Outline

**Experimental Procedures**

**Supplementary Figures and Tables**

**References**

## Experimental Procedures

### Preparation of $LaFe_{0.9}M_{0.1}O_{3-\delta}$ (M = Co, Ni, Cu) submicrofibers

The one-dimensional perovskite submicrofibers were prepared *via* the electrospinning method followed by the calcination process. Take $LaFeO_{3-\delta}$ submicrofibers as an example. Firstly, 2 mmol $La(NO_3)_3 \cdot 6H_2O$ and 2 mmol $Fe(NO_3)_3 \cdot 9H_2O$ were dissolved in 10 mL DMF by ultrasonication, where 1.1 g polyvinylpyrrolidone powder was then added slowly and stirred overnight at room temperature to mix the metal precursors and polymer chains well. The obtained metal salts-polymer solution was loaded into a plastic syringe equipped with a 23G needle for electrospinning process. The applied voltage and distance between the needle tip and the collector were fixed at 23 kV and 13 cm, respectively. The feeding rate for the precursor solution was controlled at 10 μL $min^{-1}$. An aluminum foil was wrapped on a rotating metal drum (500 rpm) as a collector. Afterward, the obtained metal salts-polymer submicrofibers were calcined under air at 700 $^{o}C$ for 5 h with a heating rate of 5 $^{o}C$ $min^{-1}$. The other perovskite submicrofibers with different compositions were prepared with a similar route, except that the stoichiometric amounts of corresponding metal nitrates were added to prepare the metal salts-polymer solutions, with a constant total amount of metal ions (4 mmol).

### Characterizations

X-ray diffraction (XRD) patterns were collected on a Bruker D8 X-ray diffractometer with a Cu Kα X-ray source (λ = 1.5418 Å). High-resolution transmission electron microscopy (HRTEM) was performed using a JEOL JEM-2100Plus electron microscope. Scanning electron microscopy (SEM) and energy-dispersive X-ray spectroscopy (EDX) mappings were carried out on a HITACHI S-4800 electron microscope. X-ray photoelectron spectroscopy (XPS) measurements and the valence band were carried out with an AXIS Supra. Inductively coupled plasma mass spectrometry (ICP-MS) was tested on a Thermo Fisher Scientific Inc ICAP TQ instrument. The ultraviolet-visible (UV-Vis) absorbance spectra were measured on TU1900 spectrophotometer. The $^{1}H$ nuclear magnetic resonance (NMR) spectra were

tested by using an AVANCE3HD600MHZ instrument. Electron paramagnetic resonance (EPR) spectra were measured at 77 K using an EPR spectrometer (Bruker A300) at 9.85 GHz. The online differential electrochemical mass spectrometry (DEMS) experiments were performed on the QAS 100 instrument. The operando Fourier transform infrared (FT-IR) spectroscopy experiments were taken with Nicolet iS50 FT-IR spectrometer. The synchrotron-based X-ray absorption spectra (XAS) of Fe K-edge were conducted at BL14W1 beamline of Shanghai Synchrotron Radiation Facility (SSRF). Fe K-edge XANES spectra were calibrated to the standard pure metal edge when performing the XAS experiments. Background subtraction and normalization were performed with the Athena software package. Wavelet transfer was performed by Matlab utilizing the source code refer to previous literature.[1,2] The k-range for the Wavelet transfer with Morlet function was selected from 0 to 14.35 $Å^{-1}$ and a k-weight of 3 was used. The Kelvin probe force microscopy (KPFM) was performed on the Hitachi AFM5100W instrument. The contact potential difference (CPD) between the tip and the sample are defined as:

$$CPD = (\varphi_{tip} - \varphi_{sample})/e$$

where $\varphi_{tip}$ and $\varphi_{sample}$ are the work function of the tip and the sample surface.

**Electrochemical measurements**

The electrochemical tests were carried out under an H-shaped electrolytic cell with a three-electrode system, where the electrolyte was 30 mL 0.5 M $Na_2SO_4$ solution with 50 ppm $NO_3^-$-N ($NO_3^-$-N represents the concentration of $NO_3^-$ expressed in terms of nitrogen content.). A perovskite-loaded carbon paper electrode, a platinum foil electrode, and a saturated Ag/AgCl electrode were used as the working electrode, counter electrode, and reference electrode, respectively. The membrane used to separate the electrolyte in the cell was a treated Nafion 117 membrane, the treatment of which was described in the previous work.[3] All potentials were converted to the potential of the relatively reversible hydrogen electrode (RHE) by the formula (E (*vs.* RHE) = E (*vs.* Ag/AgCl) + 0.197 + 0.059 × pH). Linear sweep voltammetry (LSV) curves were scanned in the range from -1.15 to -0.60 V. The potentiostatic tests were conducted at

constant potentials for 2 h. Electrochemical impedance spectroscopy (EIS) was measured at -0.9 V from 0.1 Hz to 100 kHz. Cyclic voltammetry curves were scanned under a potential window between 0.9 and 1.0 V. The double-layer capacitance ($C_{dl}$) is the slope obtained by fitting these data for sweep speed and current density at 0.95 V. The electrochemical tests were conducted using three measurements, and the performance was evaluated based on the average of these three measurements. The standard deviation of the three performance values was used as the error range.

**Cathode preparation**

Approximately 6 mg of catalyst and 60 μL of a 5 wt% Nafion solution were dispersed in 940 μL of absolute ethyl alcohol. The mixture was sonicated to achieve a homogeneous ink. Subsequently, 90 μL of the prepared ink was applied onto a carbon paper electrode (CPE) with dimensions of 1 cm × 1 cm. The electrode was then dried in an Ar atmosphere at 80 °C for 1 h.

**Determination of ammonia ($NH_3$) using the indophenol blue method**

The concentration of produced $NH_3$ in the electrolyte was spectrophotometrically determined by the indophenol blue method. In detail, 2 mL of 1 M NaOH solution containing salicylic acid (5 wt.%) and sodium citrate (5 wt.%) was added in 2 mL electrolyte. Subsequently, 1 mL of NaClO solution (0.05 M) and 0.2 mL of sodium nitroferricyanide solution (1 wt.%) were added to the above solution. Absorbance measurements were performed from 500 nm to 800 nm. The concentration-absorbance (at 655 nm) curve was calibrated using standard $NH_4^+$ solutions with a series of concentrations. The fitting curve ($y = 0.348x + 0.0134$, $R^2 = 0.999$) shows good linear relation of absorbance value with $NH_3$ concentration. Before conducting the experiments, it is essential to appropriately dilute the concentration of the electrolyte within the detectable range, where the range of dilution factors is from 4 to 10-folds.

**Determination of nitrate ($NO_3^-$)**

Firstly, 1.0 mL electrolyte was taken out from the electrolytic cell and diluted to 5.0 mL

to detection range. Then, 0.1 mL 1 M HCl and 0.01 mL 0.8 wt% sulfamic acid solution were added to the solution. After 15 min, the absorbance was detected by UV-Vis spectrophotometry at a wavelength of 220 nm and 275 nm. The final absorbance of $NO_3^-$-N was calculated based on the following equation: $A = A_{220nm} - 2A_{275nm}$. The calibration curve can be obtained through different concentrations of $NaNO_3$ solutions and the corresponding absorbance.

**Determination of nitrite ($NO_2^-$)**

20 g of *p*-aminobenzenesulfonamide was added to a mixed solution of 250 mL of water and 50 mL of phosphoric acid. Subsequently, 1 g of *N*-(1-naphthyl)-ethylenediamine dihydrochloride was dissolved in the solution. The resulting solution was then transferred to a 500 mL volumetric flask and diluted to the mark. 1.0 mL solution with different concentrations of $NaNO_2$ was diluted to 5 mL to fall within detection range. Following that, 0.1 mL color reagent was added into the above 5 mL solution. After being shaken and left to stand for 20 min, the absorbance was measured using UV-Vis spectrophotometry at a wavelength of 540 nm. By using solutions of known concentrations and their corresponding absorbance values, a calibration curve can be obtained. The nitrite concentration of the electrolyte was measured by doubling the dilution on this basis.

**Calculation of the $NH_3$ yield rate, the Faradaic efficiency (FE), and the N-selectivity of $NH_3$ and $NO_2^-$**

$NH_3$ yield rate was calculated using the following equation:

$$NH_3 \text{ yield rate} = [NH_4^+] \times V / (m_{cat.} \times t)$$

FE was calculated according to the following equation:

$$FE = 8 \times F \times [NH_4^+] \times V / (17 \times Q)$$

Where $[NH_4^+]$ is the measured $NH_4^+$ concentration, $V$ is the volume of the cathodic reaction electrolyte, $t$ is the potential applied time, $m_{cat.}$ is the loaded quality of catalyst, F is the Faraday constant, and $Q$ is the quantity of applied electricity.

The N-selectivity values of $NH_3$ and $NO_2^-$ were calculated using the following

equations:

$$\text{NH}_3 \text{ selectivity} = [NH_4^+] / \Delta[NO_3^-] \times 100\%$$

$$\text{NO}_2^- \text{ selectivity} = [NO_2^-] / \Delta[NO_3^-] \times 100\%$$

Where $\Delta[NO_3^-]$ and $[NO_2^-]$ are the concentration difference of $NO_3^-$ before and after electrolysis and the measured $NO_2^-$ concentration.

**Density functional theory (DFT) calculations**

The DFT calculations were implemented by Vienna *ab initio* simulation package (VASP),[4,5] where the ion-electron interaction was depicted by projector augmented waves (PAW) and the exchange and correlation potential was described by the revised function of Perdew, Burke and Ernzerhof (revPBE) based on the generalized gradient approximation (GGA).[6] In this calculation, a $3 \times 5 \times 1$ Gamma k-points grids is used to sample the Brillouin zone for the supercell structure. Cut-off energy of 500 eV was adopted in all calculations. The vacuum space is set to be at least 20 Å to separate the interactions between the neighboring slabs. Criteria of convergence was set to $1 \times 10^{-5}$ eV and 0.01 eV/Å for the self-consistent field (SCF) and ion steps, respectively. The free energy diagram is plotted at equilibrium potential as $\Delta G = \Delta E + \Delta ZPE - T\Delta S + eU$, where $\Delta E$, $\Delta ZPE$, T, and $\Delta S$ are the electronic energy difference, the change in zero-point energy, the temperature (T = 298.15 K), and the entropy change, respectively. As the adsorbed structures or intermediates are solid, their entropies are neglected.

**Finite element analysis**

The COMSOL Multiphysics simulation was based on a model of submicrofiber. A positive electric field was set up on the surface of submicrofiber with certain numbers of anions and cations. The domain equations can be derived by starting with the Nernst-Planck Equations. The material balance equation for the species *i* in the electrolyte was given by the continuity equation, with a flux given by the Nernst-Planck equation:

$$\partial c_i/\partial t + \nabla \cdot (\mathbf{J}_i + c_i\mathbf{u}) = R_{i,\text{tot}}$$

Where $c_i$, $t$, $\mathbf{u}$, $\mathbf{J}_i$, and $R_{i,\text{tot}}$ are the concentration of species $i$, time, velocity vector, molar flux relative to the convective transport, and source, respectively.

## Supplementary Figures and Tables

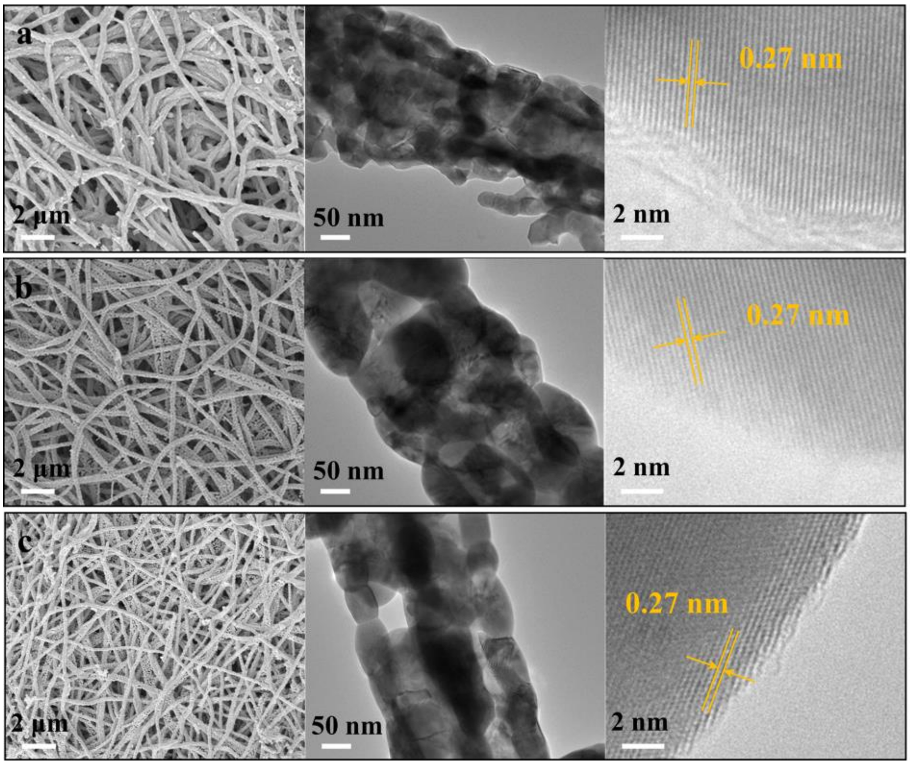


**Figure S1.** SEM, TEM, and HRTEM images of (a) LF, (b) $LF_{0.9}Co_{0.1}$, and (c) $LF_{0.9}Ni_{0.1}$ submicrofibers.

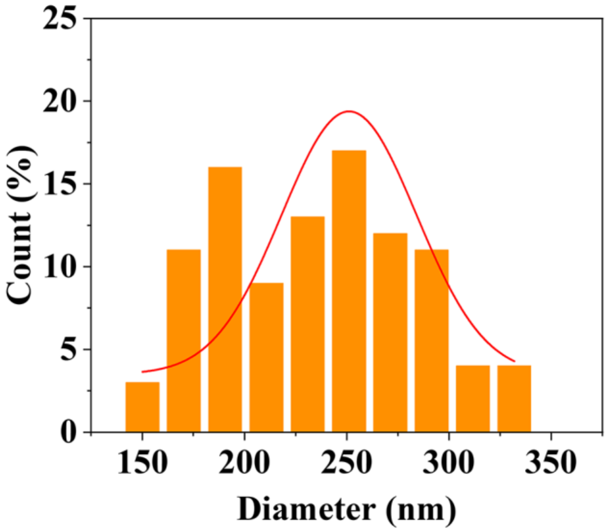


**Figure S2.** Diameter distribution of $LF_{0.9}Cu_{0.1}$ submicrofibers.

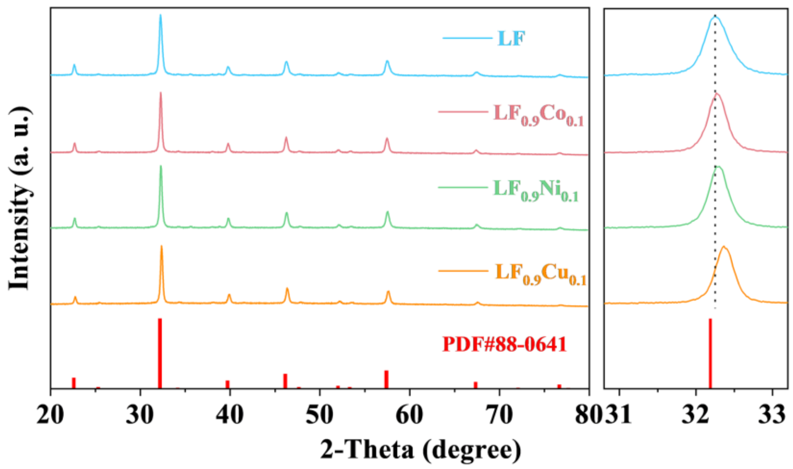


**Figure S3.** XRD patterns of LF, $LF_{0.9}Co_{0.1}$, $LF_{0.9}Ni_{0.1}$, and $LF_{0.9}Cu_{0.1}$ submicrofibers.

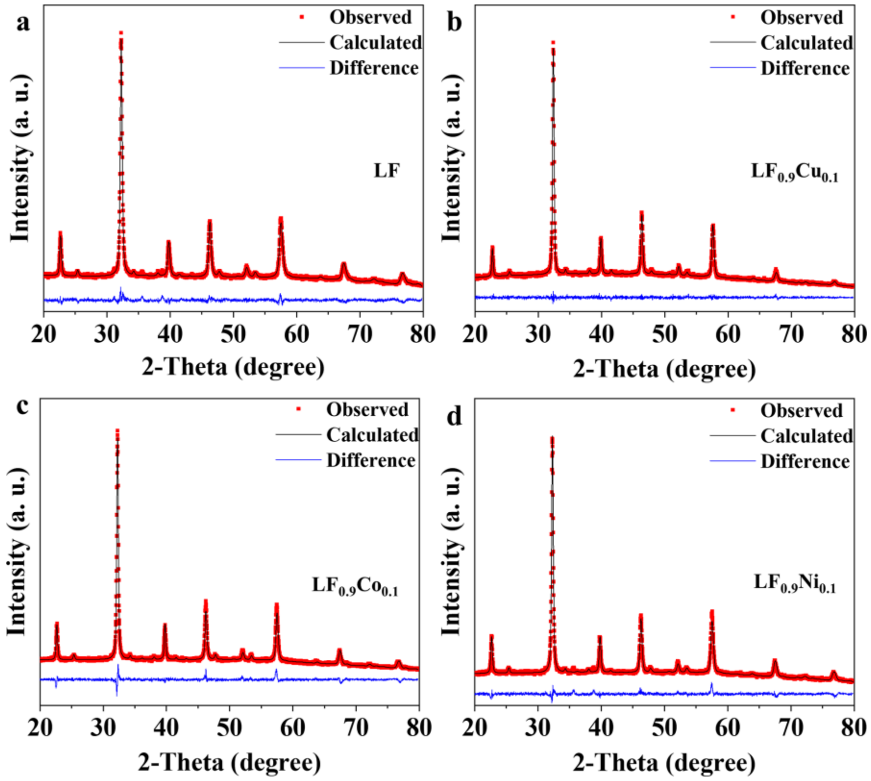


**Figure S4.** Rietveld refinement of XRD patterns of LF, $LF_{0.9}Cu_{0.1}$, $LF_{0.9}Co_{0.1}$, and $LF_{0.9}Ni_{0.1}$ submicrofibers.

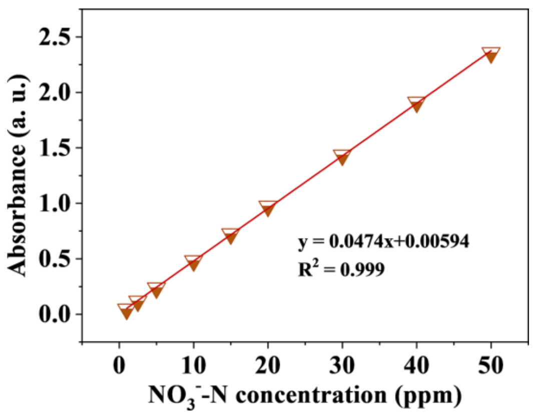


**Figure S5.** Calibration curve of $NO_3^-$-N with good linearity (the final absorbance of $NO_3^-$-N was calculated based on the following equation: $A = A_{220nm} - 2A_{275nm}$).

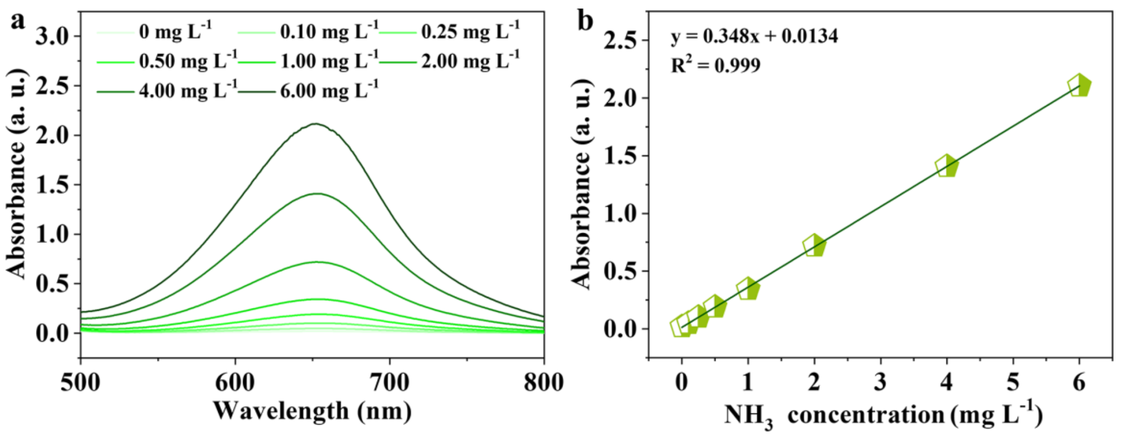


**Figure S6.** (a) UV-Vis curves and (b) corresponding calibration curve of indophenol assays with known $NH_3$ concentrations (the absorbance was measured at a wavelength of 655 nm).

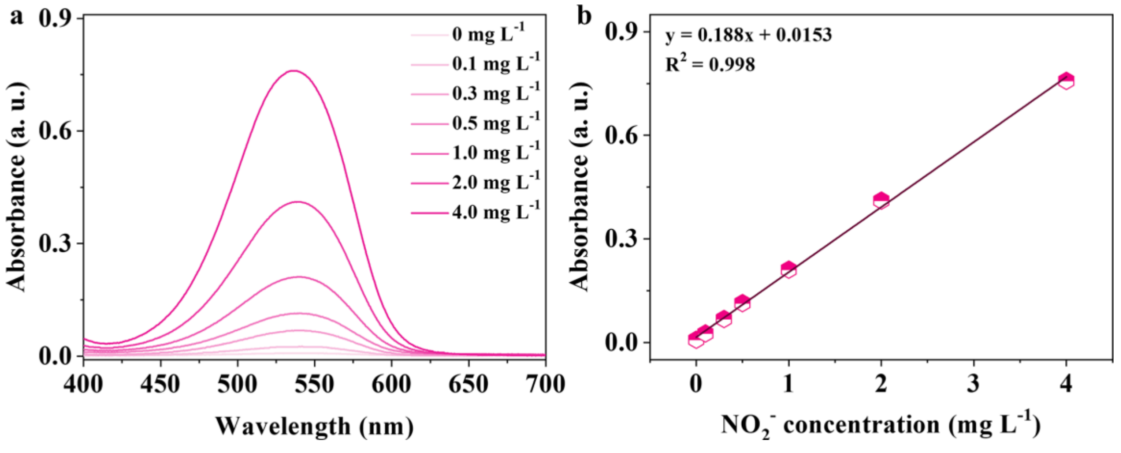


**Figure S7.** (a) UV-Vis curves and (b) corresponding calibration curve with known $NO_2^-$ concentrations (the absorbance was measured at a wavelength of 540 nm).

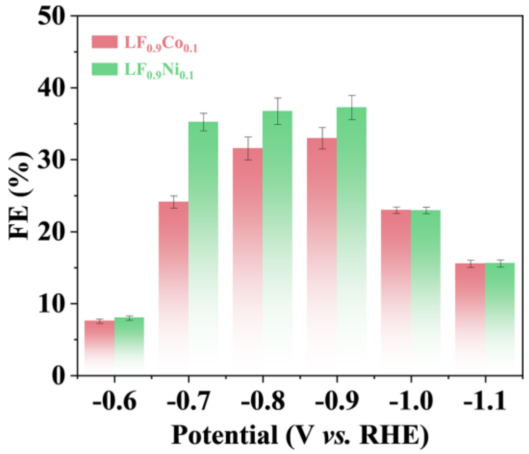


**Figure S8.** FEs of $LF_{0.9}Co_{0.1}$ and $LF_{0.9}Ni_{0.1}$ submicrofibers at given potentials.

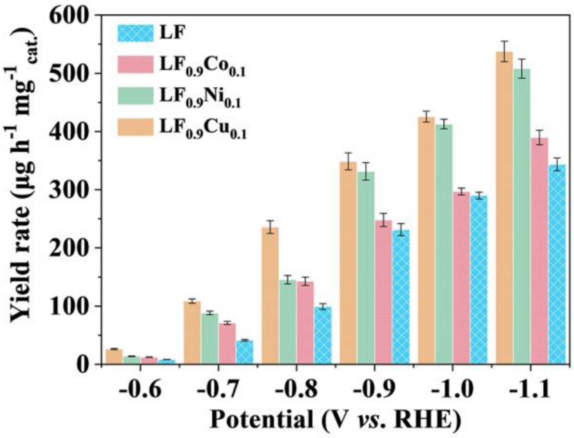


**Figure S9.** $NH_3$ yield rates of LF, $LF_{0.9}Co_{0.1}$, $LF_{0.9}Ni_{0.1}$, and $LF_{0.9}Cu_{0.1}$ submicrofibers at given potentials.

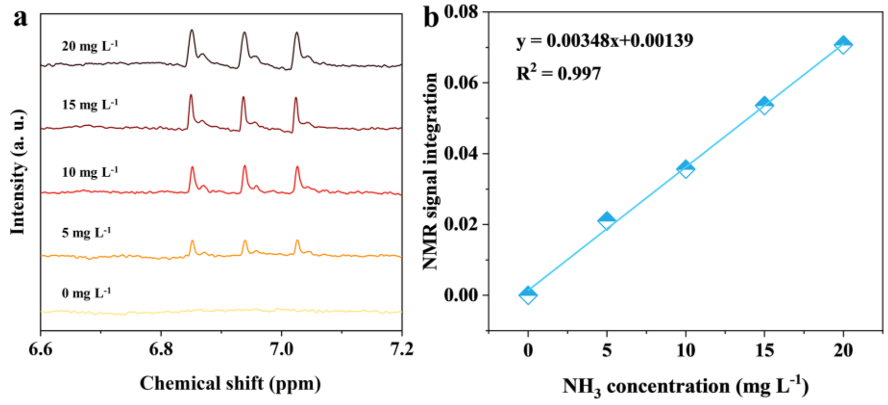


**Figure S10.** (a) $^1H$ NMR spectra of standard $(NH_4)_2SO_4$ solutions with known concentrations. (b) Concentration-integral area curve for the standard $(NH_4)_2SO_4$ solutions.

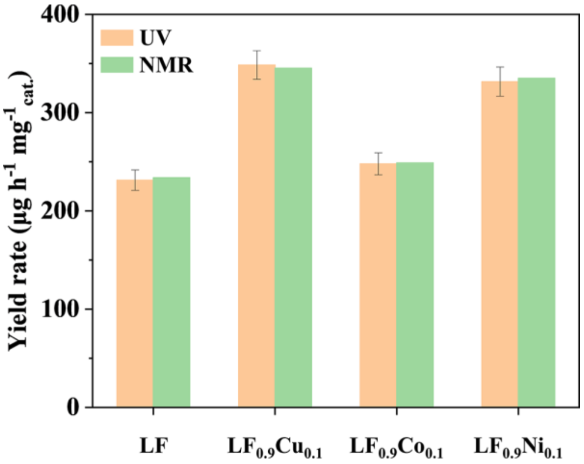


**Figure S11.** Comparison of the $^{1}$H NMR and UV-vis methods for the quantitative analysis of $NH_3$ yield rates at -0.9 V.

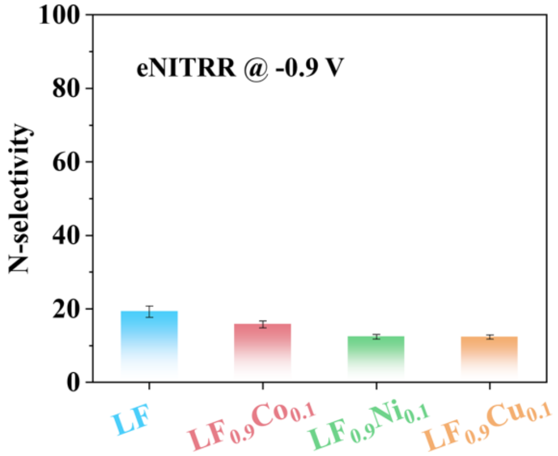


**Figure S12.** N-selectivity for $NO_2^-$ of LF and $LF_{0.9}M_{0.1}$ (M = Co, Ni, and Cu) submicrofibers at -0.9 V.

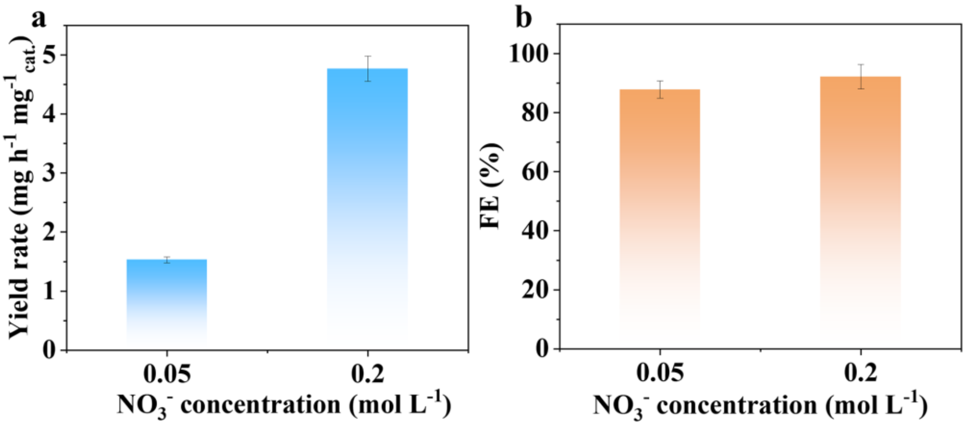


**Figure S13.** $NH_3$ yield rates and FEs of $LF_{0.9}Cu_{0.1}$ submicrofibers at different $NO_3^-$ concentrations.

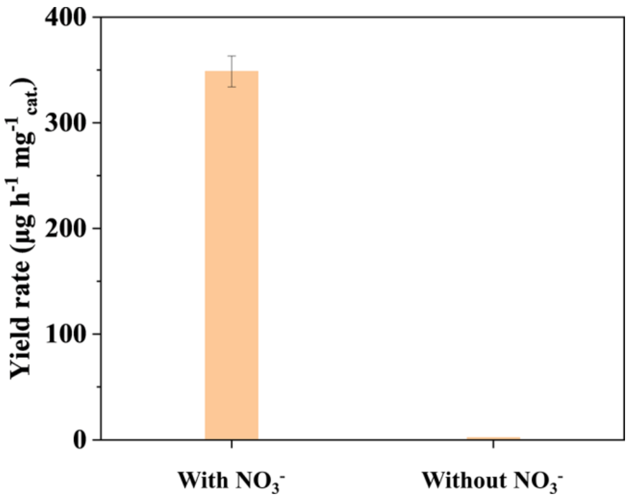


**Figure S14.** The $NH_3$ yield rates over $LF_{0.9}Cu_{0.1}$ in 0.5 M $Na_2SO_4$ electrolyte with and without $NO_3^-$ at -0.9 V.

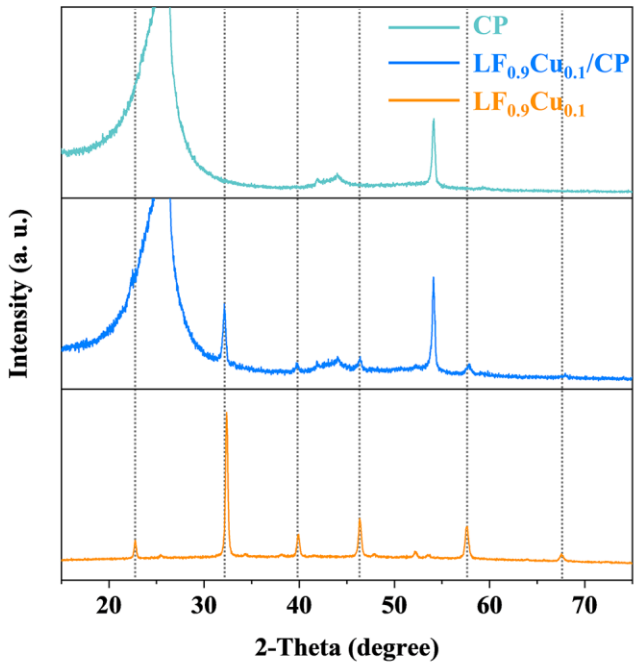


**Figure S 15.** XRD patterns of carbon paper (CP), $LF_{0.9}Cu_{0.1}$/CP after eNITRR process, and fresh $LF_{0.9}Cu_{0.1}$.

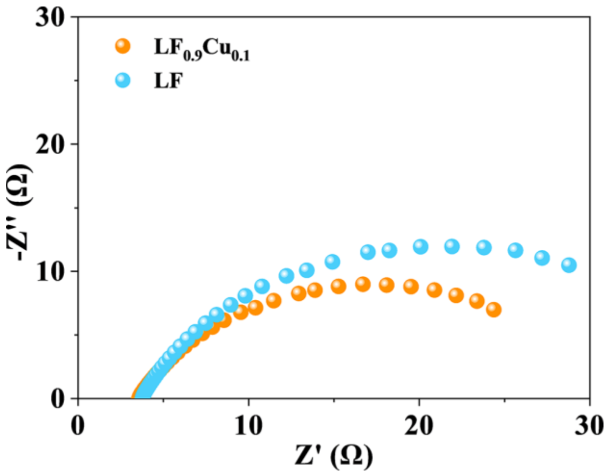


**Figure S16.** Nyquist plots for LF and $LF_{0.9}Cu_{0.1}$ submicrofibers.

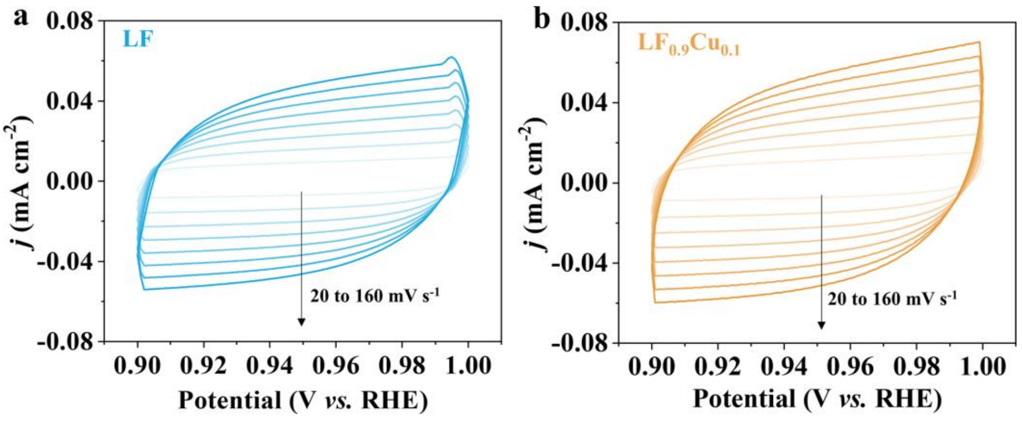


**Figure S17.** CV measurements for testing the ECSAs of (a) LF and (b) $LF_{0.9}Cu_{0.1}$ submicrofibers.

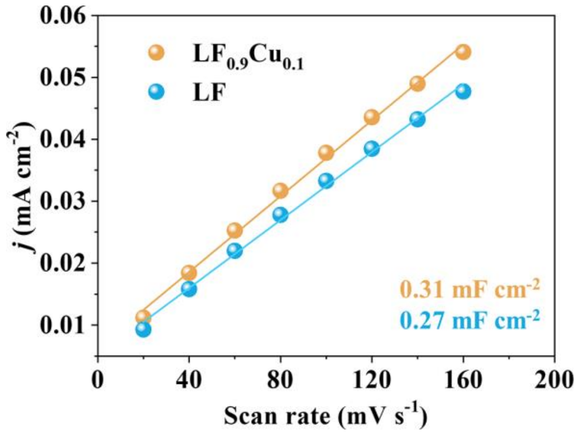


**Figure S18.** Charging current density difference plotted against scan rate of LF and $LF_{0.9}Cu_{0.1}$ submicrofibers.

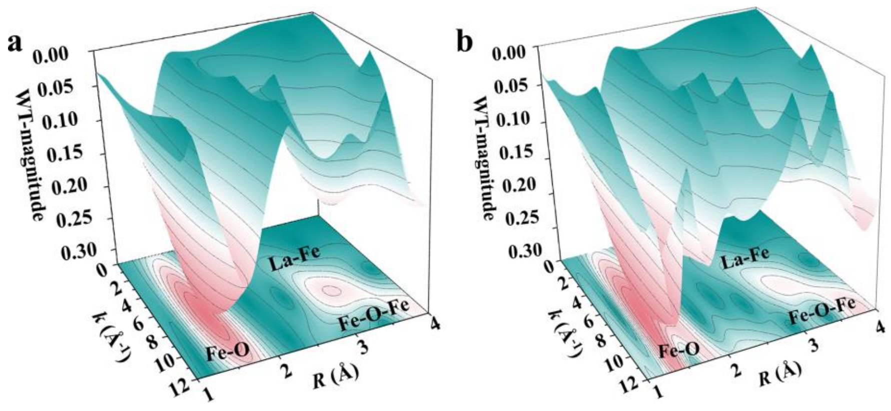


**Figure S19.** WT-EXAFS at Fe K-edge of (a) LF and (b) $LF_{0.9}Cu_{0.1}$.

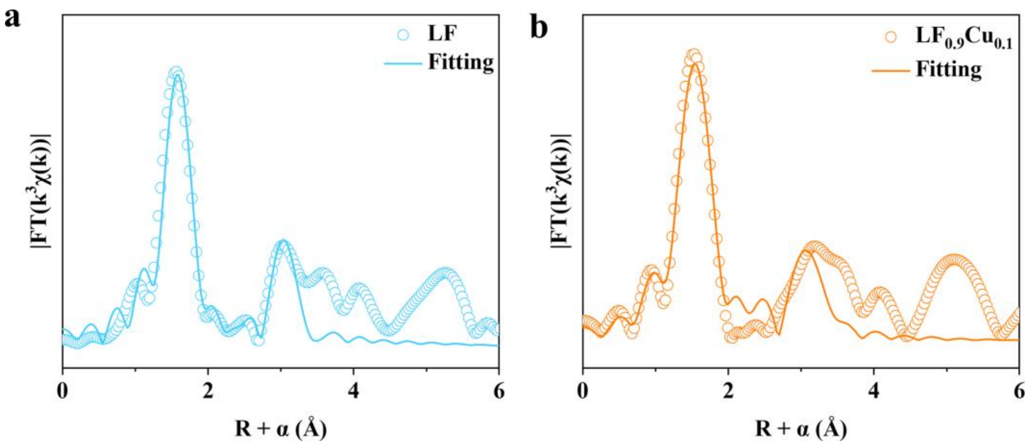


**Figure S20.** The Fe K-edge EXAFS fitting spectra of (a) LF and (b) $LF_{0.9}Cu_{0.1}$ submicrofibers in *R* space (the α on the horizontal axis represents the deviation between the experimental results and the actual bond lengths).

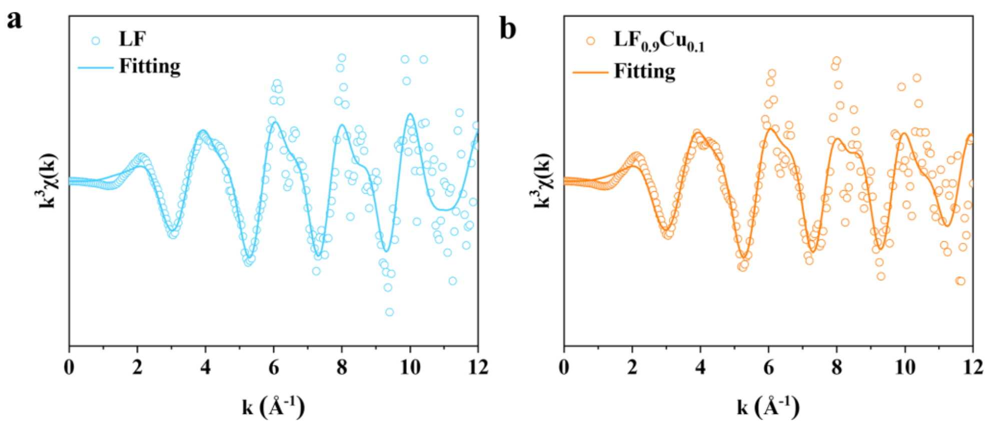


**Figure S21.** The Fe K-edge EXAFS fitting spectra of (a) LF and (b) $LF_{0.9}Cu_{0.1}$ submicrofibers in *k* space.

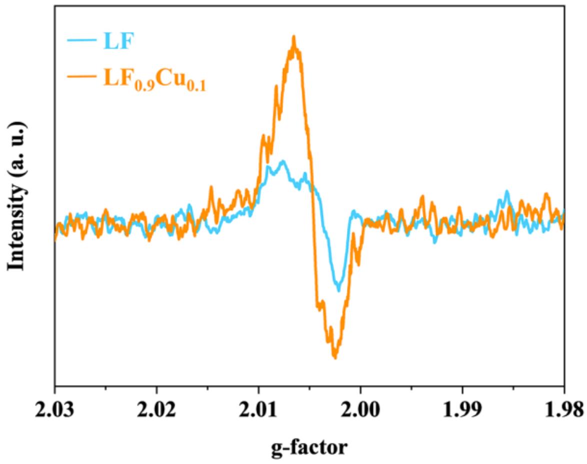


**Figure S22.** EPR spectra of LF and $LF_{0.9}Cu_{0.1}$ submicrofibers.

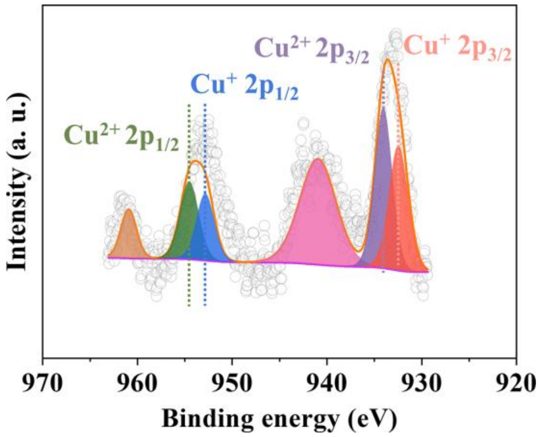


**Figure S23.** Cu 2p XPS spectrum of $LF_{0.9}Cu_{0.1}$ submicrofibers after stability test.

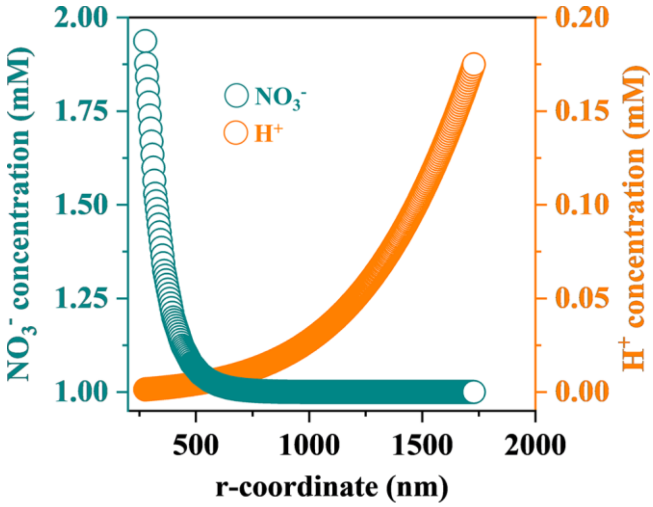


**Figure S24.** The variation curves of anion ($NO_3^-$) and cation ($H^+$) concentrations with an enhanced positive surface potential on the catalyst surface.

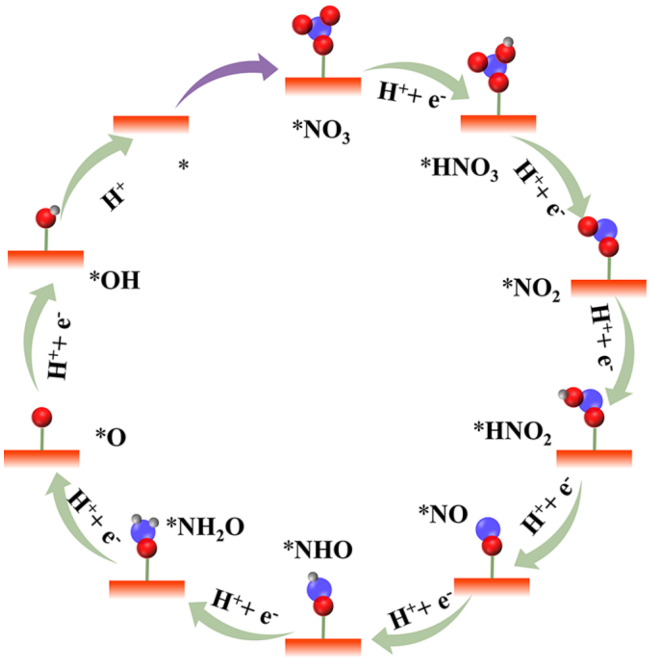


**Figure S25.** eNITRR reactive route.

**Table S1.** The refined lattice parameters of LF, $LF_{0.9}Cu_{0.1}$, $LF_{0.9}Co_{0.1}$, and $LF_{0.9}Ni_{0.1}$

| Samples | Space group | Lattice parameters (Å) | Goodness of fit |
|---|---|---|---|
| LF | Pnma | $a$ = 5.5601 Å, $b$ = 7.8688 Å, $c$ = 5.5341 Å<br>$\alpha = \beta = \gamma$ = 90°, $V$ = 242.12 Å$^3$ | $R_p$ = 4.501%<br>$R_{wp}$ = 5.789%<br>$\chi^2$ = 1.284 |
| $LF_{0.9}Cu_{0.1}$ | Pnma | $a$ = 5.5484 Å, $b$ = 7.8453 Å, $c$ = 5.5312 Å<br>$\alpha = \beta = \gamma$ = 90°, $V$ = 240.77 Å$^3$ | $R_p$ = 4.751%<br>$R_{wp}$ = 6.284%<br>$\chi^2$ = 1.047 |
| $LF_{0.9}Co_{0.1}$ | Pnma | $a$ = 5.5553 Å, $b$ = 7.8381 Å, $c$ = 5.5667 Å<br>$\alpha = \beta = \gamma$ = 90°, $V$ = 242.39 Å$^3$ | $R_p$ = 4.958%<br>$R_{wp}$ = 6.473%<br>$\chi^2$ = 1.368 |
| $LF_{0.9}Ni_{0.1}$ | Pnma | $a$ = 5.5466 Å, $b$ = 7.8579 Å, $c$ = 5.5484 Å<br>$\alpha = \beta = \gamma$ = 90°, $V$ = 241.83 Å$^3$ | $R_p$ = 4.829%<br>$R_{wp}$ = 6.293%<br>$\chi^2$ = 1.258 |

**Table S2.** Chemical composition of the synthesized samples determined by ICP-MS measurement

| Sample | Nominal composition | ICP-MS composition |
|---|---|---|
| LF | $LaFeO_{3-\delta}$ | $LaFe_{0.99}O_{3-\delta}$ |
| $LF_{0.9}Cu_{0.1}$ | $LaFe_{0.9}Cu_{0.1}O_{3-\delta}$ | $LaFe_{0.92}Cu_{0.10}O_{3-\delta}$ |
| $LF_{0.9}Co_{0.1}$ | $LaFe_{0.9}Co_{0.1}O_{3-\delta}$ | $LaFe_{0.90}Co_{0.10}O_{3-\delta}$ |
| $LF_{0.9}Ni_{0.1}$ | $LaFe_{0.9}Ni_{0.1}O_{3-\delta}$ | $LaFe_{0.91}Ni_{0.10}O_{3-\delta}$ |

**Table S3.** Comparison of $NH_3$ selectivity by electrocatalytic $NO_3^-$ reduction

| Electrocatalyst | Electrolyte | $NH_3$ Selectivity | Ref. |
|---|---|---|---|
| $Co_3O_4$-$TiO_2$/Ti | 50 ppm $NO_3^-$ + 0.1 M $Na_2SO_4$ + PVP + 1000 ppm $Cl^-$ | 24% | 7 |
| Cu/$Cu_2O$ NWAs | 200 ppm $NO_3^-$-N + 0.5 M $Na_2SO_4$ | 81.2% | 8 |
| Ni-Fe@$Fe_3O_4$ | 50 ppm $NO_3^-$ + 10 mM NaCl | 10.4% | 9 |
| $Co_3O_4$/Ti | 100 ppm $NO_3^-$-N + 0.05 M $Na_2SO_4$ | ~70% | 10 |
| Pd-Cu/γ$Al_2O_3$ | 50 ppm $NO_3^-$-N | 19.6% | 11 |
| Fe(20%)@N-C | 50 ppm $NO_3^-$-N + 50 mM $Na_2SO_4$ | < 75% | 12 |
| $La_2Cu_{0.8}Ni_{0.2}O_4$ | 50 ppm $NO_3^-$-N + 0.5 M $Na_2SO_4$ | 49.5% | 13 |
| $La_2CuO_4$ | 50 mg $L^{-1}$ $NO_3^-$-N + 0.05 M $Na_2SO_4$ | ~82 % | 14 |
| $Co_3O_4$@NiO | 200 mg $L^{-1}$ $NO_3^-$-N + 0.5 M $Na_2SO_4$ | 62.3% | 15 |
| $Co_3O_4$/CF | 50 mg $L^{-1}$ $NO_3^-$ + 0.05 M $Na_2SO_4$ | 82.1% | 16 |
| CuFe | 200 mg $L^{-1}$ $NO_3^-$-N + 0.1 M $Na_2SO_4$ | 86.8% | 17 |
| $LaFe_{0.9}Cu_{0.1}O_{3-\delta}$ | 50 ppm $NO_3^-$-N + 0.5 M $Na_2SO_4$ | 88% ± 4% | This work |

**Table S4.** Chemical composition of the $LF_{0.9}Cu_{0.1}$ (before and after stability test) determined by ICP-MS measurement

| Sample | ICP-MS composition | ICP-MS composition after stability test |
|---|---|---|
| $LaFe_{0.9}Cu_{0.1}O_{3-\delta}$ | $LaFe_{0.92}Cu_{0.10}O_{3-\delta}$ | $LaFe_{0.91}Cu_{0.08}O_{3-\delta}$ |

**Table S5.** Fe 2p XPS peak fitting parameters on LF and $LF_{0.9}Cu_{0.1}$ surfaces

LF- Fe 2p

| Name | Peak position (eV) | FWHM | Peak area |
|---|---|---|---|
| $Fe^{2+}$ $2p_{1/2}$ | 723.7 | 2.8 | 8801 |
| $Fe^{3+}$ $2p_{1/2}$ | 725.5 | 4.8 | 8548 |
| $Fe^{2+}$ $2p_{3/2}$ | 710.2 | 2.4 | 17087 |
| $Fe^{3+}$ $2p_{3/2}$ | 712.1 | 4.8 | 16866 |

$LF_{0.9}Cu_{0.1}$- Fe 2p

| Name | Peak position (eV) | FWHM | Peak area |
|---|---|---|---|
| $Fe^{2+}$ $2p_{1/2}$ | 723.7 | 3.0 | 7305 |
| $Fe^{3+}$ $2p_{1/2}$ | 725.5 | 4.9 | 9361 |
| $Fe^{2+}$ $2p_{3/2}$ | 710.2 | 2.4 | 14384 |
| $Fe^{3+}$ $2p_{3/2}$ | 712.1 | 4.9 | 18848 |

**Table S6.** O 1s XPS peak fitting parameters and the concentrations of oxygen vacancies on LF and $LF_{0.9}Cu_{0.1}$ surfaces

LF-O 1s, $(O_2^{2-}/O^-)\% = 13.6\%$

| Name | Peak position (eV) | FWHM | Peak area |
|---|---|---|---|
| Lattice $O^{2-}$ | 528.8 | 1.7 | 35264 |
| $O_2^{2-}/O^-$ | 530.4 | 1.6 | 5543 |
| Surface-adsorbed $O_2$ or hydroxyl groups | 531.4 | 1.4 | 15387 |
| Surface-adsorbed $H_2O$ | 532.7 | 1.9 | 8862 |

$LF_{0.9}Cu_{0.1}$-O 1s, $(O_2^{2-}/O^-)\% = 17.6\%$

| Name | Peak position (eV) | FWHM | Peak area |
|---|---|---|---|
| Lattice $O^{2-}$ | 528.8 | 1.3 | 27510 |
| $O_2^{2-}/O^-$ | 530.4 | 1.6 | 5862 |
| Surface-adsorbed $O_2$ or hydroxyl groups | 531.4 | 1.4 | 15607 |
| Surface-adsorbed $H_2O$ | 532.7 | 1.9 | 7618 |

**Table S7.** Cu 2p XPS peak fitting parameters on $LF_{0.9}Cu_{0.1}$ surface

| $LF_{0.9}Cu_{0.1}$- Cu 2p | | | |
|---|---|---|---|
| Name | Peak position (eV) | FWHM | Peak area |
| $Cu^{+}$ $2p_{1/2}$ | 952.8 | 1.8 | 2039 |
| $Cu^{2+}$ $2p_{1/2}$ | 954.5 | 3.5 | 3256 |
| $Cu^{+}$ $2p_{3/2}$ | 932.4 | 1.8 | 4052 |
| $Cu^{2+}$ $2p_{3/2}$ | 934.0 | 3.7 | 6154 |

**Table S8.** Cu 2p XPS peak fitting parameters on $LF_{0.9}Cu_{0.1}$ surfaces after stability test

| $LF_{0.9}Cu_{0.1}$- Cu 2p | | | |
|---|---|---|---|
| Name | Peak position (eV) | FWHM | Peak area |
| $Cu^{+}$ $2p_{1/2}$ | 952.8 | 2.2 | 2529 |
| $Cu^{2+}$ $2p_{1/2}$ | 954.5 | 2.4 | 3097 |
| $Cu^{+}$ $2p_{3/2}$ | 932.4 | 2.4 | 5082 |
| $Cu^{2+}$ $2p_{3/2}$ | 934.0 | 2.2 | 6005 |

**References:**

1. Muñoz, M.; Farges, F.; Argoul, P. Continuous Cauchy wavelet transform of XAFS spectra. *Phys. Scr.* **2005**, *115*, 221-222.

2. Muñoz, M.; Argoul, P.; Farges, F. Continuous Cauchy wavelet transform analyses of EXAFS spectra: A qualitative approach. *Am. Mineral.* **2003**, *88*, 694–700.

3. Han, L.; Liu, X.; Chen, J.; Lin, R.; Liu, H.; Lü, F.; Bak, S.; Liang, Z.; Zhao, S.; Stavitski, E.; Luo, J.; Adzic, R. R.; Xin, H. L. Atomically dispersed molybdenum catalysts for efficient ambient nitrogen fixation. *Angew. Chem. Int. Ed.* **2019**, *58*, 2321–2325.

4. Kresse, G.; Furthmüller, J. Efficient iterative schemes for ab initio total-energy calculations using a plane-wave basis set. *Phys. Rev. B* **1996**, *54*, 11169–11186.

5. Kresse, G.; Hafner, J. Ab initio molecular dynamics for open-shell transition metals. *Phys. Rev. B* **1993**, *48*, 13115–13118.

6. Kresse, G.; Furthmüller, J. Efficiency of ab-initio total energy calculations for metals and semiconductors using a plane-wave basis set. *Comput. Mater. Sci.* **1996**, *6*, 15–50.

7. Gao, J.; Jiang, B.; Ni, C.; Qi, Y.; Zhang, Y.; Oturan, N.; Oturan, M. A. Non-precious $Co_3O_4$-$TiO_2$/Ti cathode based electrocatalytic nitrate reduction: Preparation, performance and mechanism. *Appl. Catal. B Environ.* **2019**, *254*, 391–402.

8. Wang, Y., Zhou, W., Jia, R., Yu, Y.; Zhang, B. Unveiling the activity origin of a copper-based electrocatalyst for selective nitrate reduction to ammonia. *Angew. Chem. Int. Ed.* **2020**, *59*, 5350–5354.

9. Jonoush, Z. A., Rezaee, A.; Ghaffarinejad, A. Electrocatalytic nitrate reduction using $Fe^0$/$Fe_3O_4$ nanoparticles immobilized on nickel foam: Selectivity and energy consumption studies. *J. Clean. Prod.* **2020**, *242*, 118569.

10. Su, L.; Li, K.; Zhang, H.; Fan, M.; Ying, D.; Sun, T.; Wang, Y.; Jia, J. Electrochemical nitrate reduction by using a novel $Co_3O_4$/Ti cathode. *Water Res.* **2017**, *120*, 1–11.

11. Zhang, Z.; Xu, Y.; Shi, W.; Wang, W.; Zhang, R.; Bao, X.; Zhang, B.; Li, L.; Cui, F. Electrochemical-catalytic reduction of nitrate over Pd–Cu/$\gamma Al_2O_3$ catalyst in cathode chamber: Enhanced removal efficiency and $N_2$ selectivity. *Chem. Eng. J.* **2016**, *290*,

201–208.

12. Duan, W.; Li, G.; Lei, Z.; Zhu, T.; Xue, Y.; Wei, C.; Feng, C. Highly active and durable carbon electrocatalyst for nitrate reduction reaction. *Water Res.* **2019**, *161*, 126–135.

13. Gong, Z.; Zhong, W.; He, Z.; Jia, C.; Zhou, D.; Zhang, N.; Kang, X.; Chen, Y. Improving electrochemical nitrate reduction activity of layered perovskite oxide $La_2CuO_4$ via B-site doping. *Catal. Today* **2022**, *402*, 259–265.

14. Yang, W.-J.; Yang, L.-H.; Peng, H.-J.; Lv, S.-H.; Sharif, H. M. A.; Sun, W.; Li, W.; Yang, C.; Lin, H. Perovskite oxide $LaMO_{3-\delta}$ (M = Fe, Co, Ni and Cu) cathode for efficient electroreduction of nitrate. *Sep. Purif. Technol.* **2022**, *295*, 121278.

15. Wang, Y., Liu, C., Zhang, B.; Yu, Y. Self-template synthesis of hierarchically structured $Co_3O_4$@NiO bifunctional electrodes for selective nitrate reduction and tetrahydroisoquinolines semi-dehydrogenation. *Sci China Mater.* **2020**, *63*, 2530–2538.

16. Fu, W., Du, X., Su, P., Zhang, Q.; Zhou, M. Synergistic effect of Co (III) and Co (II) in a 3D structured $Co_3O_4$/carbon felt electrode for enhanced electrochemical nitrate reduction reaction. *ACS Appl. Mater. Interfaces* **2021**, *13*, 28348–28358.

17. Wang, C.; Liu, Z.; Hu, T.; Li, J.; Dong, L.; Du, F.; Li, C.; Guo, C. Metasequoia-like nanocrystal of iron-doped copper for efficient electrocatalytic nitrate reduction into ammonia in neutral media. *ChemSusChem* **2021**, *14*, 1825–1829.
